# Functional metagenomics identifies novel genes *ABCTPP, TMSRP1* and *TLSRP1* among human gut enterotypes

**DOI:** 10.1038/s41598-018-19862-5

**Published:** 2018-01-23

**Authors:** Manoj Kumar Verma, Vasim Ahmed, Shashank Gupta, Jitendra Kumar, Rajesh Pandey, Vibha Mandhan, Nar Singh Chauhan

**Affiliations:** 1 Department of Biochemistry, Maharshi Dayanand University, Rohtak, Haryana India; 2 Ayurgenomics Unit-TRISUTRA, Council of Scientific and Industrial Research-Institute of Genomics and Integrative Biology, New Delhi, India; 3 Present Address: Mammalian Genetics Unit, MRC Harwell Institute, Harwell Science and Innovation Campus, Oxfordshire, OX11 0RD United Kingdom

## Abstract

Every niche in the biosphere is touched by the seemingly endless capacity of microbes to transform the world around them by adapting swiftly and flexibly to the environmental changes, likewise the gastrointestinal tract is no exception. The ability to cope with rapid changes in external osmolarity is an important aspect of gut microbes for their survival and colonization. Identification of these survival mechanisms is a pivotal step towards understanding genomic suitability of a symbiont for successful human gut colonization. Here we highlight our recent work applying functional metagenomics to study human gut microbiome to identify candidate genes responsible for the salt stress tolerance. A plasmid borne metagenomic library of *Bacteroidetes* enriched human fecal metagenomic DNA led to identification of unique salt osmotolerance clones SR6 and SR7. Subsequent gene analysis combined with functional studies revealed that *TLSRP1* within *pSR*7 and *TMSRP1* and *ABCTPP* of *pSR*6 are the active loci responsible for osmotolerance through an energy dependent mechanism. Our study elucidates the novel genetic machinery involved in bestowing osmotolerance in *Prevotella* and *Bacteroidetes*, the predominant microbial groups in a North Indian population. This study unravels an alternative method for imparting ionic stress tolerance, which may be prevalent in the human gut microbiome.

## Introduction

Microbes are ubiquitous in nature and have been identified to exist in all potential habitats. Microbial adaptation occurs primarily through genetic evolution, in response to various environmental stressors, which is clearly reflected in the successful microbial presence across a wide range of ecosystems, such as hot springs, salt brines, alkaline lakes and acid mine drainage^1,2^. The majority of the microbes growing under salt stress conditions were reported to be osmotolerant for a short duration to even their entire life span^3^. Mechanisms responsible for high salt stress tolerance include induction of stress tolerance proteins, enhanced activity of Na^+^/H^+^ pumps, modifications in membrane composition and an ability towards increased uptake of compatible solutes^3,4^. A variety of studies have been carried out to identify the gene or gene cluster responsible for salt tolerance in different culturable bacteria^5–7^. However, there are a large number of unculturable bacteria, which are expected to possess various novel physiological mechanisms conferring salt tolerance^8,9^. Metagenomics is a cultivation-independent genome-level characterization of communities and their members inhabiting a particular environmental niche^10,11^ and further, functional metagenomics has been utilized to identify various novel genes and physiological pathways, which are prevalent in diverse ecosystems^12,13^. The human gut microbiome has become perhaps the most intensively studied environment using metagenomics for decoding the physiological role of gut microbes in human health^14,15^. Studies have highlighted various important insights, but there are potentially large proportion of genes which remains uncharacterized. Microbial diversity within the human gut plays a significant role in human health, physiology and tissue development^16^. They do so by their ability to counter numerous environmental stresses such as low pH, bile acids, elevated osmolarity, iron limitation, insufficient nutrient availability and host immune factors^17–19^. These physiological mechanisms which cumulatively resist rapid changes in the gastrointestinal environment are crucial for their successful colonization^19,20^. Efforts were made to elucidate salt tolerance genes from human gut microbiome and following salt tolerance genes including *brp/blh, galE, murB*, *mazG* were identified^21–23^. Prevalence of only a few osmotolerance genes within a highly diverse human gut microbiome seems unrealistic and thus offers scope for further identification of novel osmotolerance mechanisms. With the advent of both faster and more accurate next-generation sequencing (NGS) technologies and functional metagenomics, together they allowed us in gaining new insights into gut microbial community structure. It also led to the discovery of three such novel osmotolerance genes i.e. *ABCTPP*, *TMSRP1* and *TLSRP1*. This study enabled us to build a strong foundation to explore various unique mechanisms of salt stress tolerance involved in the human gut microbiome.

## Results

### Phylogenetic reconstruction of human gut microbiomes

**Figure 1:**
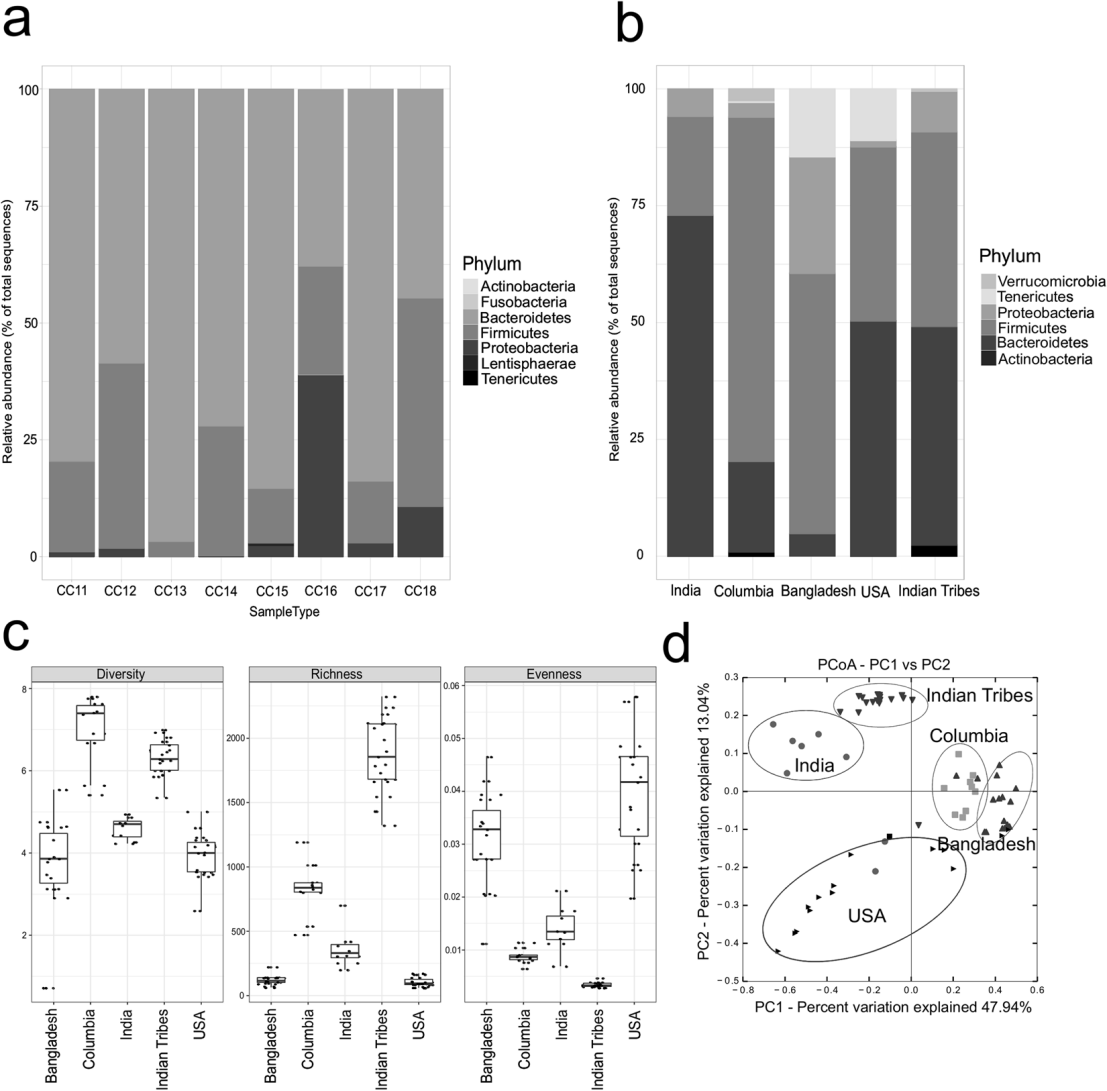
Analysis of Human gut microbiome structure. Taxonomic profiles of the gut microbiota among Indian samples (**a**), Taxonomic profiles of the gut microbiota among different populations (**b**), Diversity estimates in gut microbial communities Diversity (Shannon), Richness, and Evenness (**c**) Principal Coordinate Analysis (PCoA) of bacterial community structures of the gut microbiota of different populations based on weighted unifrac distance metrics (**d**).

The SSU rRNA gene sequence analysis yielded 1,69,185 quality checked sequences for the studied samples (Table S1). Greengene based OTU identification through closed reference OTU picking protocol of QIIME resulted in the identification of 1453 unique OTUs. Taxonomic summary shows that the majority of the OTUs were affiliated to Bacteroidetes (65.25%), Firmicutes (22.85%) and Proteobacteria (8.66%) (Fig. 1a). However *Prevotella* genus (64.94%) was found to be dominating among Bacteroidetes, whereas *Lachnospiraceae* family (42.30%) was dominant among Firmicutes.

We analyzed the differences between gut microbiota of India, Bangladesh, USA and Columbia populations (Table S2). We obtained in total 57,17,311 sequences which contributed to 8193 OTUs. Taxonomic summary among different populations is shown in (Fig. 1b,c). A significantly different human gut microbiome structure was observed within studied populations. The relationships between the community structures among different populations were examined using the Principal Coordinate Analysis (PCoA) based on the weighted unifrac distance matrixes (Fig. 1d). PCoA plot clearly shows that the Indian population is different from Columbia, USA and Bangladesh, even though Bangladesh population shares most of the microbiota with Indian population as seen in earlier studies^24^. Based on LDA effect size (LEfSe), 101 bacterial taxas were significantly more abundant between India, Columbia, USA, Indian tribal and Bangladesh populations. Amidst, 19 bacterial taxas in USA, 21 taxas in India, 20 taxas in Columbia, 15 taxas in Indian tribes and 26 taxas in Bangladesh were differentially present (Table S3). All these taxas belong to Bacteroidetes, Firmicutes, Proteobacteria and Tenericutes phyla. Among all, *Prevotella copri* is found to be significantly abundant (*P* = value < 0.05) in Indian population (Table S3).

### Screening and characterization of salt resistant clones

**Figure 2:**
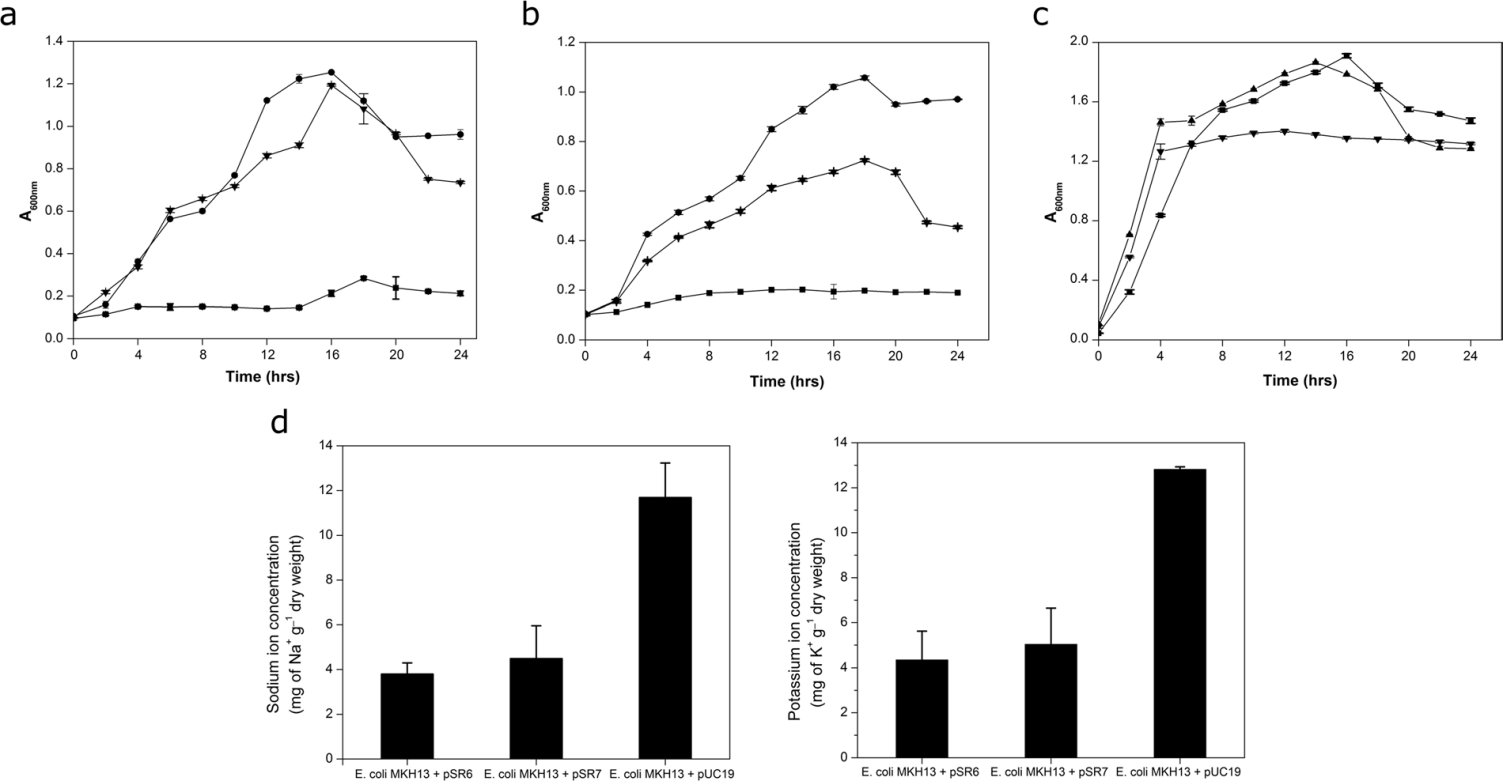
Growth curve analysis of clones. Growth of *E.coli* (MKH13) metagenomic clones harboring *pSR*6 (●), *pSR*7 (▼) and *E.coli* (MKH13) host strain carrying empty plasmid vector (■) in, (**a**) LB broth supplemented with 3.0% NaCl (w/v), (**b**) LB broth supplemented with 4% KCl (w/v), (**c**) LB broth. Intracellular K^+^ and Na^+^ estimation in *E.coli* (MKH13) clones harboring *pSR*6 and *pSR*7 and *E.coli* (MKH13) host strain carrying empty plasmid vector (**d**). Each point in graph is the mean of three different replicate experiments, each performed in triplicate.

Metagenomic DNA of highly diverse sample (CC16) was used for construction of a metagenomic library, representing 1,69,148 recombinant plasmid clones. Initial screening of CC16 metagenomic library led to identification of nine salt tolerant clones. Subsequent RFLP analysis indicated the presence of two unique recombinant clones, hereby referred as SR6 and SR7. The SR6 and SR7 have a statistically significant growth advantage in the presence of 3.5% (w/v) NaCl (*P* = 0.0005 and *P* = 0.0004) and 5.0% (w/v) KCl (*P* = 0.000006; *P* = 0.00002), respectively as compared to *E*. *coli* (DH10B) strain carrying empty plasmid vector (Figure S1a and b). A non-significant difference in growth pattern of control and test clones were observed in the absence of salt stressors (*P* = 0.0490; *P* = 0.0490) (Figure S1c). Minimum inhibitory concentration analysis of SR6 and SR7 showed significant growth up to 4.0% (w/v NaCl) (*P* = 0.0158; *P* = 0.0344) and 6.7% (w/v KCl) (*P* = 0.0099; *P* = 0.0088) as compared to control *E*. *coli* (DH10B) (Figure S2a and b). Complementation analysis with *pSR*6 and *pSR*7 has shown a statistically significant growth advantage to the salt sensitive *E*. *coli* (MKH13) host in the presence of 3.0% (w/v) NaCl (*P* = 0.00005 and *P* = 0.00002) and 4.0% (w/v) KCl (*P* = 0.00003; *P* = 0.00002), respectively as compared to salt sensitive *E*. *coli* (MKH13) strain carrying empty plasmid vector (Fig. 2a,b). A non-significant difference in growth pattern of the test and control was observed in the absence of salt stressors (*P* = 0.41946; *P* = 0.07151) (Fig. 2c). Elemental analysis showed that clone SR6 and SR7 has maintained a significantly lower intracellular concentration of Na^+^ (*P* = 0.0205 and *P* = 0.0411) and K^+^ (*P* = 0.0112 and *P* = 0.0210) in the presence of salt stressors (NaCl (3% w/v) and KCl (4% w/v) in comparison to the *E*. *coli* MKH13 strain (Fig. 2d).

### Genetic characterization of salt tolerance genes

The plasmid insert for *pSR*6 and *pSR*7 were sequenced and assembled to generate a sequence of 1313 bp and 1915 bp respectively. Further, G + C content of *pSR*6 and *pSR*7 was found to be 52.54% and 43.23% respectively. A nucleotide BLAST of *pSR*6 and *pSR*7 inserts showed a homology of 89% and 99% with uncultured bacterial clones from fecal sample of Crohn’s disease patient (EU064107). Additionally, gene prediction server indicated the presence of two complete *ORF*s (*ORF*1 and 2) in *pSR*6, encoding inner membrane bound protein of 332aa and 213aa respectively (Table 1). Translated nucleotide sequences were subjected to BLASTP (maximum e-value cutoff of 1e-34) analysis to identify homologous sequence in the NCBI database (Table 1). The pblast database homologs of translated *pSR*6 *ORF*1 corresponds to putative membrane transporter proteins of *Prevotella species*, while *ORF*2 has no significant similarity. Furthermore, *ORF*1 and *ORF*2 were in reverse orientation and overlapping in nature. Transposon mutagenesis analysis identified a functionally active locus within the sequence (621 bp and 979 bp), which encompasses *ORF*1 as well as *ORF*2. It indicates that possibly both *ORFs* are working either individually or cooperatively to provide salt resistance to the host.

**Table 1:** Genes identified in the present study.

Clone plasmid	Putative gene	ORF location	Database homolog	% identity
*pSR6* (1313)	*ABCTPP*	260–901 (+2)	No Match	Nil
*pSR6* (1313)	*TMSRP1*	1308–310 (−3)	Putative ABC transporter permease protein *of* *Prevotella copri* CAG:164	94
*pSR7* (1915)	*TLSRP1*	61–1914 (+1)	Putative uncharacterized protein of *Bacteroides sp*. CAG:875	62

The G + C content of *ORF*1 is 53.42% and Pfam analysis of *ORF*1 encoded protein, speculates it as a putative ABC transporter permease having conserved domains for MacB-like periplasmic core domain and FtsX-like permease family. Further, STRING functional assignment of *ORF*1 encoded protein predicted it as an ABC transporter containing transmembrane domains (TMD) and ATP Binding domains (NBD). The predicted NBD is known to generate energy for transport of a number of toxic molecules through TMD^25^. HMMTOP analysis has predicted three transmembrane helices within *ORF*1 at 202–224; 255–274 and 295–318, possibly synthesizing its TMD. Additionally, NsitePred has identified strong nucleotide binding sites (predominantly ATP Binding) between 59–60 and 104–107 of *ORF*1. These nucleotide binding sites could be NBD, as predicted by STRING functional assignment. To summarize, *ORF*1 encodes an ATP binding cassette (ABC) transporter permease protein (ABCTPP), hence *ORF*1 is labelled as a putative *ABCTPP* gene.

On the other hand, G + C content of *ORF*2 is 56.55% and share no homology within NCBI, pfam and STRING database. An upstream ribosomal binding site at −8 to −10 position and an upstream promoter element (−33) were predicted, indicating its possibility as an independent novel *ORF*. The encoded protein of *ORF*2 harbors a signal peptide (1–33) with five transmembrane helices. RaptorX binding prediction server has predicted binding sites for organic molecules (Glycerol, 2,3-Dihydroxy-1,4-Dithiobutane 1,2-Ethanediol, AMP and UMP) and ions (potassium, sulfate etc.) within its domain. As *ORF*2 has been identified to play a significant role in salt tolerance it is described as a Transmembrane Salt Resistance Protein 1 gene (*TMSRP1 gene*) encoding *TMSRP1*.

The *pSR7* sequence indicated the presence of single *ORF* encoding cytosolic protein of 617 amino acids that shared 62% homology with uncharacterized protein of *Bacteroidetes sp*. Functional assignment with Pfam- and STRING database search indicates presence of *TonB* dependent receptor plug domain, with a possible function to sense the environment outside the cell and allow direct movement of substances (macromolecules, small molecules and ions) across cell by means of some agent such as a transporter, pore or a motor protein^26^. This is an A + T rich sequence (G + C: 42.98%), encoding a cytosolic protein having a number of nucleotide binding sites (GTP: 36, ATP:15). Transposon mutagenesis also confirmed functionally active locus within this *ORF*. The phylogenetic analysis further identifies it as a member of *TonB* linked outer membrane protein sub-family. Hereby, cumulating its physiological role and predicted features, this gene was described as TonB linked Salt Resistance Protein 1 gene (TLSRP1 gene) encoding *TLSRP1*.

### Physiological characterization of salt tolerance genes

**Figure 3:**
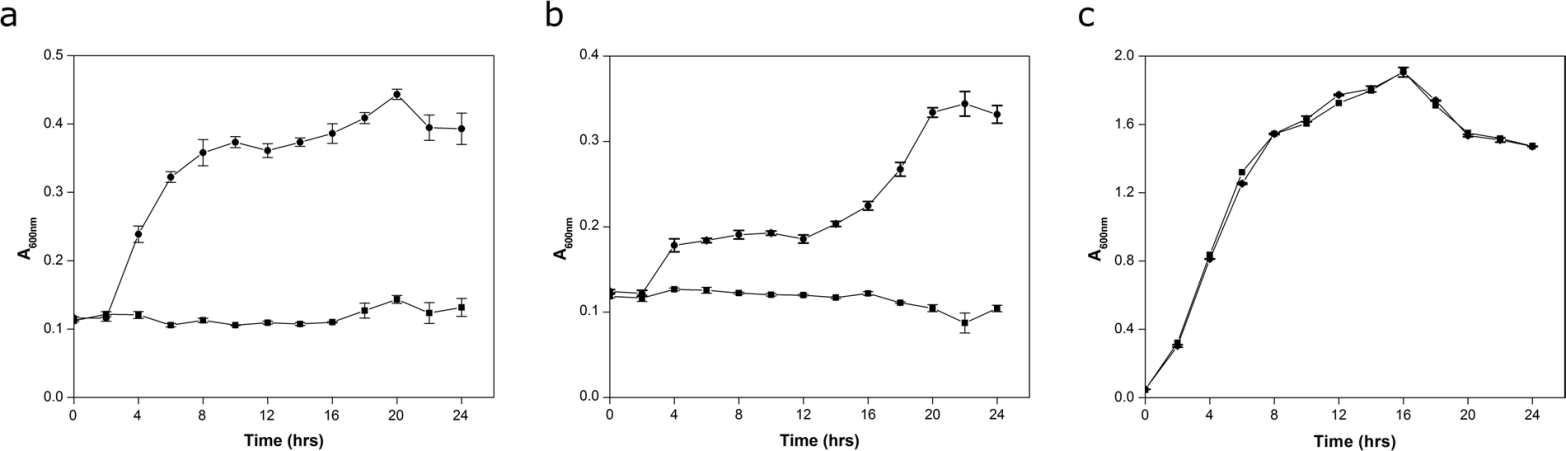
Characterization of *ABCTPP* for osmotolerance. Growth curve analysis of osmotolerant phenotype SR6C1 (●) and *E.coli* (MKH13) host strain carrying empty plasmid vector (■) in (**a**) LB broth supplemented with 2% NaCl (w/v), (**b**) LB broth supplemented with 4% KCl (w/v), and (**c**) LB only. Each point in graph is the mean of three different replicate experiments, each performed in triplicate.

**Figure 4:**
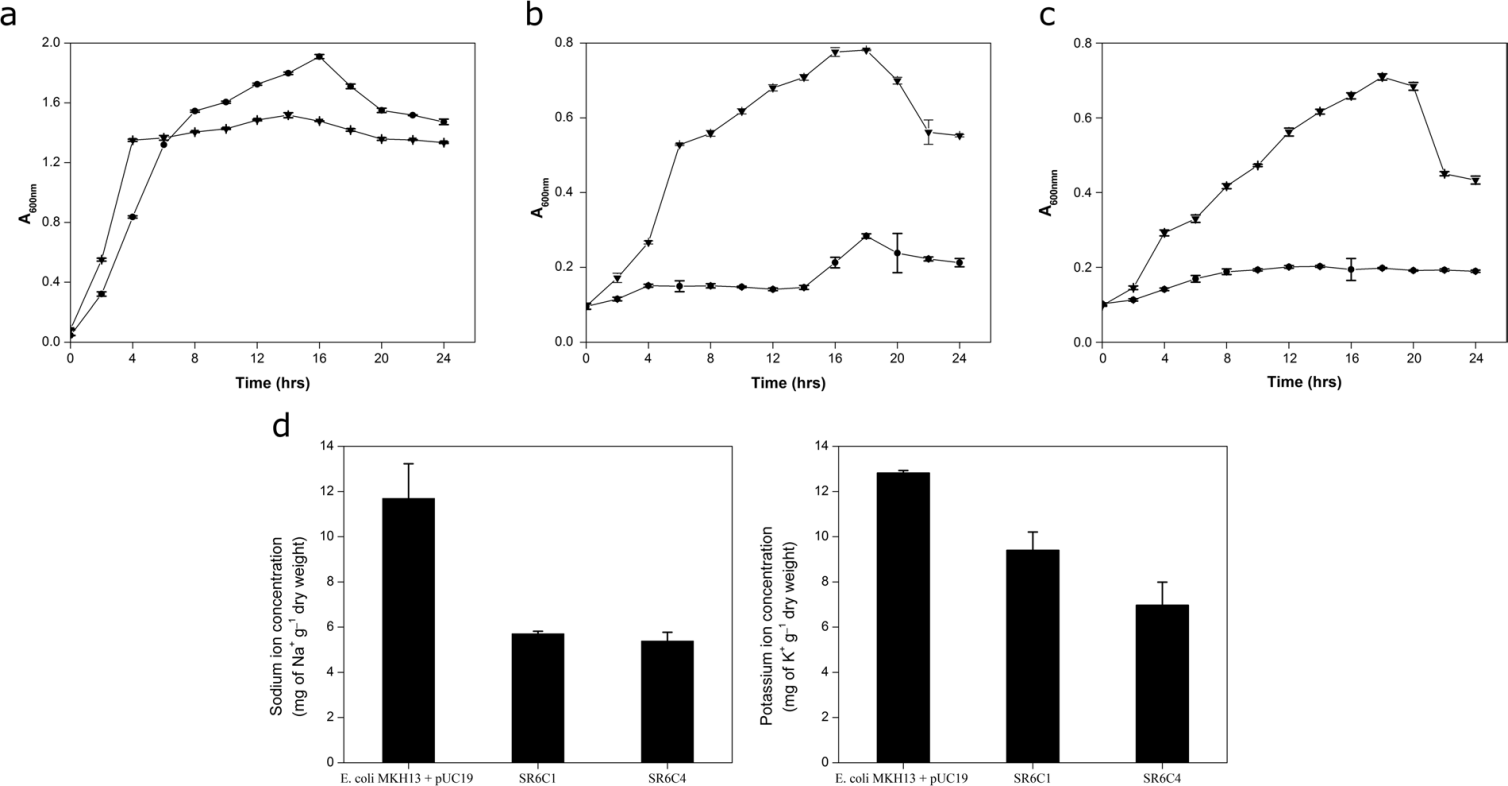
Characterization of *TMSRP1* for osmotolerance. Growth of osmotolerant phenotype SR6C4 (■) and *E.coli* (MKH13) host strain carrying empty plasmid vector (●) in (**a**) LB broth, (**b**) LB broth supplemented with 4.0% KCl (w/v), and (**c**) LB broth supplemented with 3% NaCl (w/v). Intracellular K^+^ and Na^+^ estimation in SR6C1 and SR6C4 and *E.coli* (MKH13) host strain carrying empty plasmid vector (**d**). Each point in graph is the mean of three different replicate experiments, each performed in triplicate.

*ABCTPP* and *TMSRP1* genes were subcloned into *pUC*19 (*pSR*6C1 and *pSR*6C3) to validate the physiological role of these genes towards host survivability in the presence of ionic stressors. Recombinant clone SR6C1 has shown significantly (*P* = 0.00103 and *P* = 0.00002) increased growth advantage in the presence of NaCl (2.0% (w/v)) and KCl (4% (w/v)) with reference to the *E*. *coli* (MKH13) having native *pUC*19 only (Fig. 3a,b). SR6C1 showed no significant growth difference when it was grown in LB only, as compared to control (Fig. 3c). Similarly, statistically significant (*P* = 0.00005 and *P* = 0.0005) growth was observed for the clone SR6C3 in reference to control *E*. *coli* (DH10B) in growth medium supplemented with NaCl (4.0% (w/v)) and KCl (5% (w/v)) individually (Fig. S3a and S3b). Minimum inhibitory concentration analysis of SR6C3 showed significant growth upto (4.0% (w/v)) NaCl and (6.7% (w/v)) KCl (*P* = 0.0057; *P* = 0.0103) as compared to control *E*. *coli* (DH10B). SR6C4 showed a significantly (*P* = 0.00006 and *P* = 0.00001) increased salt tolerance as compared to *E*. *coli* (MKH13) host carrying the empty vector only, when grown in NaCl (3% (w/v)) and KCl (4% (w/v)) individually (Fig. 4a,b), while no significant growth difference (*P* = 0.19462 and *P* = 0.07151) was observed in growth medium lacking salt stresses (Fig. 4c). Elemental analysis showed that *E*. *coli* (MKH13) clone harboring *pSR*6C1 and *pSR*6C3 significantly lowered their intracellular concentration of Na^+^ (*P* = 0.0440 and *P* = 0.0343) and K^+^ (*P* = 0.0275 and *P* = 0.0152) in the presence of salt stressors NaCl (3% w/v) and KCl (4% w/v) individually in comparison to the *E*. *coli* (MKH13) strain (Fig. 4d).

*In silico* analysis of these genes has indicated the requirement of energy rich molecules like ATP for extending host osmotolerance. Thus, we selected a couple of compounds to check whether it is true for our putative gene functions. Dicyclohexylcarbodiimide (200 μM), an F0 F1-ATPase inhibitor and sodium orthovanadate (2 mM), a P-type ATPase inhibitor remarkably diminished the growth of SR6C1, SR6C3 and SR7 clones in the presence of 4% (w/v) NaCl and 5% (w/v) KCl individually. However, valinomycin (1 mM), affected the growth of SR6C1, SR6C3 and SR7 only in the presence of 5% (w/v) KCl. These results clearly demonstrate that *TLSRP1* within *pSR*7 and *TMSRP1* and *ABCTPP* of *pSR*6 extends host osmotolerance through an energy (ATP) dependent process.

### Transcriptional regulation of TMSRP1 gene

**Figure 5:**
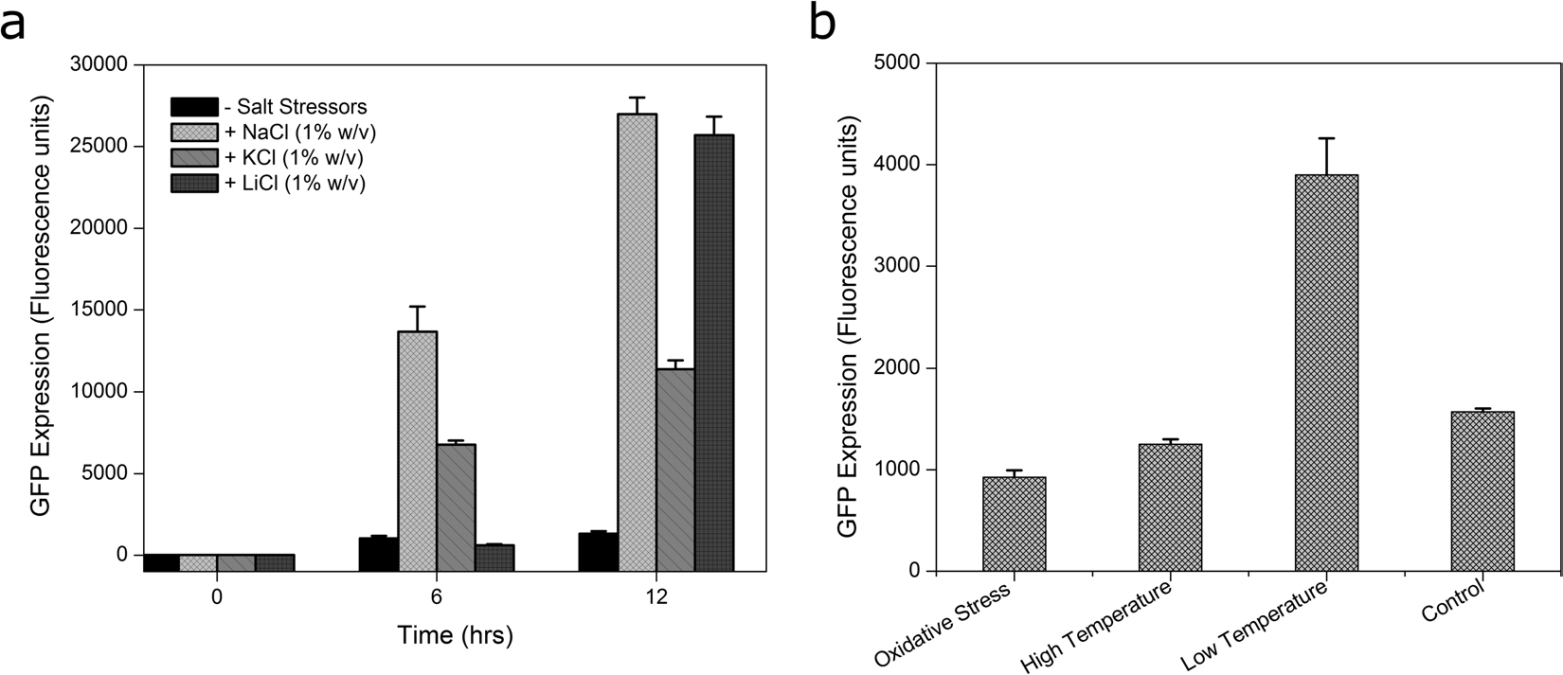
Transcriptional regulation of *TMSRP1*. GFP expression of recombinant clone PR1 phenotype with (**a**) 1% (w/v) NaCl, KCl and LiCl, 100 mM Hydrogen peroxide and at various temperatures 15 °C, 37 °C and 45 °C (**b**). Each point is the mean of three different replicate experiments, each performed in triplicate.

The predicted upstream promoter region of *TMSRP1* was cloned upstream of promoterless green fluorescent protein (GFP) gene in the broad host range vector *pPROBE-GT* (Figure S4). Presence of osmotic stressors has significantly increased GFP expression in PR1 (Fig. 5a,b), while no significant change was observed in other stress conditions (Fig. 5c). Although even at low temperature (15 °C), GFP expression of pPR1 was found to be significantly elevated.

### Gene abundance of TMSRP1 and other genes

The HMP database which contains all metagenomic datasets encompassing sixteen body sites from the Human Microbiome Project (HMP) was also screened for *TMSRP1, ABCTPP* and *TLSRP1* homologs. Hits to *TMSRP1, ABCTPP* and *TLSRP1* were most abundant in the stool, supragingival plaque and left retro-auricular crease metagenome samples at the lowest e-value (Figure S5a, b and c). Additionally, *TMSRP1, ABCTPP* and *TLSRP1* showed homology with and *Bacteroidetes*/*Prevotella* species during NCBI database search (Figure S5d, e and f).

## Discussion

In the current study, we highlight our recent work applying functional metagenomics to study human gut microbiome to identify candidate genes responsible for the salt stress tolerance and discuss how an approach combining high-throughput sequencing, cultivation and metagenomic functional screens can improve our understanding of interactions between microbes and its human host under adverse salt conditions. In present study, the human gut microbiome structure of the North Indian subject, elucidated the dominance of *Bacteroidetes*/*Prevotella* phylotype. Functional metagenomics, transposon mutagenesis and bioinformatics strategies were used to identify novel salt tolerance genes (*TMSRP1*, *ABCTPP* and *TLSRP1*) from the human gut microbiota. The presence of these genes in human gut microbes is likely to be important in the human gut and further identification of these genes would also help in elucidating the unique mechanism of salt tolerance involved in the human gut microbiome^8,10^.

SSU rRNA sequences identified that the Bacteroidetes dominate gut of studied subjects, which were known to play vital role in physiological processes of digestion, host immunity, host interaction, supply of nutrients and degradation of xenobiotics^15,16,27–29^. The abundance of Bacteriodetes, especially *Prevotella* sp. within various population, including Indian population, had already been deciphered from a number of human gut microbiome studies^16,24,27,30^. Dominance of *Prevotella* in Indian subjects could be associated with dietary habits^24^. *Prevotella*/*Bacteroides* are known for their metabolic potential to utilize the complex polysaccharides, as substrate^24,31^. It might provide an advantage over other gut residents for successful colonization and dominance in the human gut. In north/west Indian populations, complex polysaccharides (wheat flour, sorghum flour and maize flour) are major dietary constituents^32^, which was utilized as a preferred metabolic substrate by *Prevotella sp*. and *Bacteriodes sp*. to release short chain fatty acids (SCFA)^24,30,31^. However, evidence for the mechanism of successful survivability of microbes in highly fluctuating and challenging environment of the gut is lacking. Presence of any solute transport gene system or unique osmotolerance system is highly anticipated within these microbial groups for their survival in the gut^22^. Identification of salt tolerant clones SR6 and *pSR*7 and more strikingly, phylogenetic similarity of *pSR*6 with *Prevotella copri* CAG:164 are interesting. Sequence analysis and transposon mutagenesis analysis have identified the bioactive locus within these clones. Sequence analysis of *pSR*6 identified presence of two overlapping but oppositely oriented salt responsive genes, not so uncommon among microbes^16,27^. Surprisingly, both these predicated genes occupied bioactive region for salt tolerance, as deciphered through transposon mutagenesis. Among them, ORF1 has shared a good homology with putative ABC transporter permease proteins of *Prevotella sp*., annotated as *ABCTPP* (ABC Transport permease protein). The physiological characterization of this gene has indicated its role in extending osmotolerance property to the host *E*. *coli* strain, possibly by reducing the intracellular concentration of ionic stressors through an energy dependent mechanism. Though a detailed mechanistic role of this gene in osmoltolerance still needs to be established, however osmotolerance through energy dependent expulsion of ionic stressors are well documented^16^. Human microbiome database search has identified an abundance of *ABCTPP* protein homologs in the human gut microbiome (stool sample), as compared to other body sites. Similarly, *Prevotella sp*. were more abundant within human gut microbiome (stool samples) as compared to other body sites^16,27^. Similarly, taxonomic distribution analysis of its homologs lies within *Bacteroides*/*Prevotella* clades. It strengthens our view on the role of *ABCTPP* gene in the maintenance of osmotic stress within *Prevotella sp*., a predominant human gut microbe.

The putative *TMSRP1* gene of *pSR*6 shares similar topology of being inner membrane bound transmembrane protein. Interestingly, alternative initiation codon of *TMSRP1* i.e. TTG other than ATG, is the common feature of osmotolerance genes in a number of gastrointestinal pathogens^33,34^. Even the presence of salt responsive promoter upstream of *TMSRP1* gene could acts as a regulator of the general stress response, induced during stationary phase in *E*. *coli* and other Gram negative bacteria^35^. It provides a strong possibility that this gene could play a significant role in host adaptability in the presence of ionic stressors. The finding is strengthened by subcloning, energy dependency and elemental analysis studies, where *TMSRP1* gene significantly improves host growth in the presence of ionic stressors by reducing their intracellular ion concentration to the host physiological limits. pSR6 encoded *TMSRP1* proteins were found to provide tolerance toward ionic stressors through energy dependent mechanism and an upstream promoter identifies its salt responsive expression. Presence of promoter has enhanced the efficiency of *TMSRP1* protein towards host tolerance against ionic stressors. The promoter was identified to respond to osmotic stressors like NaCl, KCl and LiCl and enhance the expression of downstream gene through many folds. Similarly, many bacteria, including *Escherichia coli* have *OxyR*, *SoxRS*, and *RpoS* as regulators of the oxidative stress^36^. Physiological, *in silico* and regulation studies demonstrate the role of *TMSRP1* and *ABCTPP* gene in host survivability during osmotic stress. However, their mechanistic role in providing salt stress tolerance still needs to be established.

Genetic and physiological characterization of salt tolerance clone *pSR*7 has identified a TonB linked outer membrane protein sub-family member and a *Bacteriodetes* homolog *TLSRP1* for osmoltolerance property. InterPro database information indicates that these family proteins allow passage of ligands using energy (proton-motive force) and through conformational alterations^37^. Members of this family are restricted to the Bacteroidetes lineage (except for *Gemmatimonas aurantiaca* T-27 from the novel phylum Gemmatimonadetes) and occur in high copy numbers, with over 100 members from *Bacteroides thetaiotaomicron* VPI-5482 alone^38^. A taxonomic affiliation of TLSRP1 gene has similar findings showing its phylogenetic lineages within *Bacteroidetes*. Published descriptions of members of this family are available for Rag A from *Porphyromonas gingivalis*^39^, SusC from *Bacteroides thetaiotaomicron*^40^, and OmpW from *Bacteroides caccae*^38^. Transporter complexes, including these outer membrane proteins are likely to import large degradation products of proteins (e.g. Rag A) or carbohydrates (e.g. Sus C) as nutrients, rather than siderophores (transport, binding proteins and unknown substrate)^39,40^. However, no information has been gathered for the role of *TonB* in osmotic stress maintenance, except our current study having deciphered role of this protein in the salt stress maintenance though a widely unknown mechanism.

## Conclusion

In this study, the functional metagenomics approach was used to decipher osmotolerant genes prevalent among one of the most abundant microbial lineages present in the human gut microbiome. Identification of *TMSRP1, ABCTPP* and *TLSRP1* genes has enriched our understanding about their survival and acclimatization in the highly variable gut ecosystem. The identified salt tolerance genes might be used as a stepping stone in the fields of patho-biotechnology and meta-biotechnology for designing improved probiotic cultures with greater resistance to induced stresses as well as improved gut colonization. Our results will further build a foundation for future studies to understand salt tolerance mechanisms involved in this unique environment.

## Materials and Methods

### Human fecal sample collection and processing

Fecal samples from eight healthy individuals not having any past history of prolonged illness, recent antibiotic usage and proactive food intake were collected in sterile containers (Table S4). The samples were then further processed for metagenomic DNA isolation^41^.

### Ethical Statement

Recruitment of volunteers and sample collection was carried out using standard procedures following ethical guidelines of Indian Council of Medical Research, India for biomedical research and informed consent of volunteers. The study has been conducted after ethical clearance from human ethical committee at M. D. University, Rohtak, Haryana, India.

### Bacterial strains and growth conditions

Bacterial strains and plasmids used in the study are listed in (Table 2). Oligonucleotide primers used in the study (GeNoRime, Shrimpex Biotech services Pvt. Ltd. India) are presented in (Table S5). *Escherichia coli* (DH10B) was cultured in Luria-Bertani (LB) medium. *E*.*coli* (DH10B) containing *pUC*19 vector were cultured in the presence of ampicillin (100 µg ml^−1^), whereas, *pPROBE-GT* promoter less vector was cultured in LB supplemented with gentamycin (10 µg ml^−1^).

**Table 2:** Bacterial strains and plasmids used in the present study.

Strains, plasmids, clones and transposons	Genotype or Characteristics	Source or reference
*E. coli* (DH10B)	F- endA1 recA1 galE15 galK16 nupG rpsL ΔlacX74 Φ80lacZΔM15 araD139 Δ(ara,leu)7697 mcrA Δ(mrr-hsdRMS-mcrBC) λ-	Invitrogen, Thermo Scientifc USA
*E. coli* (MKH13)	MC4100Δ(putPA)101D(proP)2D(proU)	^56^
*pSR6*	*pUC19* based harbouring a metagenomic DNA fragment of 1313 bp having osmotolerance property	Present work
*pSR7*	*pUC19* based harbouring a metagenomic DNA fragment of 1915bp having osmotolerance property	Present work
SR6	*pSR6* in *E. coli* DH10B host	Present work
SR6	*pSR7* in *E. coli* DH10B host	Present work
*pSR6C1*	*pUC19* recombinant plasmid harboring *ABCTPP* gene of pSR6 cloned at *EcoR1* and *HindIII of pUC19 MCS (Multiple cloning site)*	Present work
SR6C1	pSR6C1 in *E. coli* MKH13 host	Present work
*pSR6C3*	*pUC19* recombinant plasmid harbouring *TMSRP1* gene with promoter of pSR6 cloned at *EcoR1* and *HindIII* of *pUC19* MCS	Present work
SR6C3	pSR6C3 in *E. coli* DH10B host	Present work
SR6C4	pSR6C3 in *E. coli* MKH13 host	Present work
*pPR1*	*pPROBE-GT* recombinant plasmid harboring predicated upstream promoter region (+35) of *TMSRP1* of pSR6 cloned at *EcoR1* site of MCS	Present work
PRI	pPR1 in *E. coli* DH10B host	Present work
*pUC19*	Plasmid cloning vector Amp^r^	Thermo Scientific
*pPROBE-GT*	Promoter less vector	Addgene, USA
Transposon, EZ-Tn5TM <Kan-2>	Tn5^TM^ Transposon Kan^r^	Epicentre Biotechnologies Madison, Wisconsins, USA

### Phylogenetic reconstruction of human gut microbiome

Metagenomic DNA was used as a template for the amplification of small subunit ribosomal RNA (SSU rRNA) gene^11^. HPLC-purified primers targeting the V1–V4 regions of the SSU rRNA gene were designed for pyrosequencing^42^. Amplified product was then used for next generation sequencing (NGS) with the aid of Roche 454 GS FLX+ platform^11,42^. Quantitative Insights Into Microbial Ecology (QIIME) 1.9.1 pipeline was implemented for pyrosequencing data analysis^43^. Chimeric sequences were detected and removed using usearch61^44^. 16S rRNA gene sequence data was curated for quality, length and ambiguous bases as a quality filtering step. Each sample was preprocessed to remove sequences with length less than 200 nucleotides and more than 1000 nucleotides sequences, with minimum average quality less than 25 and containing >2 ambiguous bases. Reads were assigned to operational taxonomic units (OTUs) using a closed reference OTU picking protocol using QIIME 1.9.1, with uclust (Edgar, 2010) being applied to search sequences against a subset of the GreenGenes database, version 13_8 filtered at 97% sequence identity. Furthermore, the alpha diversity (Shannon diversity and observed species) for all samples was calculated using QIIME, to estimate species diversity, richness and evenness, and visualized using ggplot2. Furthermore, overall taxonomic differences and beta diversity were estimated through Principal Coordinates Analysis (PCoA) based on weighted unifrac distances.

### Comparative analysis of human gut microbiomes

We used publically available 16S rRNA gene sequence data for human gut from different populations^45,46^ to see any determining microbial diversity among populations^24^ (Table S2). To avoid the biasness introduced due to respective studies describing microbiota of these populations, sequence data of individuals from a study was merged and considered as a representative microbiota of that country^24^. Raw data from all these samples were processed along with the in-house samples in the same way as explained earlier. Linear discriminant analysis coupled with effect size (LEfSe) was performed to identify the bacterial taxa differentially represented between higher taxonomy levels^47^. For bacterial groups, the LDA score threshold was set to >4 whereas for functional genes and their specific KEGG orthologs, the LDA score threshold was set to >2.5.

### Screening and characterization of salt resistant clones

A plasmid borne human gut metagenomic library was prepared^13,48^ from CC16 and used to screen for salt tolerant clones^22^. A total of 1,69,148 clones were screened on LB agar supplemented with 4.3% (w/v) NaCl. Plasmid isolation, restriction enzyme digestion, ligation and competent cell preparation were carried out using standard procedures^49^. The minimum inhibitory assay and growth inhibition studies of clones SR6 and SR7 were performed for indexing their osmotolerance property^22^. Graphs (created using Origin61) were presented as an average of triplicate experiments, with error bars being representative of the standard error of the mean (SEM). Further the p value was calculated using t-test available with Origin61software.

### Genetic and physiological characterization of salt tolerance genes

The plasmid insert from *pSR*6 and *pSR*7 was sequenced using Sanger sequencing chemistry with primer walking approach at Eurofins Genomics India Pvt Ltd (Bangalore, India). Sequence assembly was performed with Seq-Man sequence assembly software of Lasergene package, version 5.07 (DNA Star, USA). Putative open reading frame (ORF) was predicted using an ORF finder tool at NCBI (http://www.ncbi.nlm.nih.gov/gorf) and checked for the database homology with Basic Local Alignment and Search Tool (BLAST) (http://www.ncbi.nlm.nih.gov/ Blast.cgi). Functional annotations of the proteins were performed with Pfam database^50^ and the STRING database^51^. The promoter was predicted with Fruitfly promoter search tool (http://www.fruitfly.org/seqtools/promoter.html). Nucleotide binding sites were predicted with Nsite Pred Server (http://biomine.cs.vcu.edu), whereas, ligand binding sites were predicted using RaptorX binding prediction server (http://raptorx.uchicago.edu/). The topology of encoded proteins was checked with HMMTOP^52^. Further, presence of signal peptide within the encoded sequence was observed with TOPCONS^53^.

Transposon mutagenesis was carried out on *pSR*6 and *pSR*7 using EZ-Tn5^TM^ <Kan-2> Insertion kit (Epicenter Biotechnologies) following manufacturer’s instructions. Transposon mutants of *pSR*6 and *pSR*7 were screened for the salt stress resistant and sensitive phenotypes to identify the functional genomic regions within the cloned DNA fragment in *pSR*6 and *pSR*7. Putative *ABCTPP*, *TMSRP1* of *pSR*6 recombinant plasmid were subcloned using standard cloning techniques^47^. The Minimum Inhibitory assay and growth inhibition studies of subclones were performed to analyze for their salt stress maintenance property^22^. Energy dependency of *ABCTPP, TMSRP1* and *TLSRP1* for salt stress tolerance were checked by growth inhibition studies in the presence of salt stress and various inhibitors^54^. All assays were performed in triplicates for calculation of standard deviation. The p value was calculated with paired t-test available with Origin61.

### Elemental quantification of Na^+^ and K^+^ in salt tolerant clones

Elemental quantification of Na^+^ and K^+^ in *E*. *coli* MKH13 carrying the empty vector (*pUC*19) and *E*. *coli* MKH13 harboring *pSR*6, *pSR*7, *pSR*6C1 and *pSR*6C3 was measured with inductively coupled plasma spectroscopy-atomic emission spectroscopy (ICP-AES) analysis at SAIF, IIT Bombay, India, in the *E*. *coli* cultures grown in the presence of ionic stressors (NaCl (3% w/v) and KCl (4% w/v), individually)^55^. Results were expressed as mg of Na^+^ g^−1^ dry weight and K^+^ g^−1^ dry weight of cells. The t-test was used for statistical analysis.

### Transcriptional regulation of TMSRP1 gene

The predicted promoter region of *TMSRP1* was amplified from pSR6 plasmid DNA with Pro fwd and Pro1 rev primer (Table S2). The promoter region was subcloned at *Eco*R1 *& Hind*III site within MCS of *pPROBE-GT* using standard molecular cloning techniques. *E*.*coli* (DH10B) carrying *pPROBE* constructs were grown in GFP medium (EDTA 0.05%, Glycerol 0.5%, Dextrose 0.5%, Orthophosphoric acid 0.2%, Ammonium sulfate 0.05%, Tris base 1.21%, Yeast Extract 0.20%, Vitamin B1 0.1%, Osmotic stressor 1% and pH 7.5) supplemented with gentamycin (10 µg ml^−1^) for 24 h. The overnight grown cultures were diluted to a final optical density of 0.1 (A_600 nm_) in fresh GFP medium to test GFP (green fluorescent protein) gene expression with low temperature (15 °C), high temperature (45 °C), 100 mM H_2_O_2_ and osmotic stress NaCl, KCl and LiCl 1% (w/v). GFP expression was checked by reading fluorescence (excitation filter at 485 nm and emission filter at 528 nm) in 1 ml aliquots at different intervals (0, 12 and 24 h) using Fluorometer (Waters USA). All experiments were performed in triplicates.

### Gene Abundance analysis

*ABCTPP, TMSRP1* and *TLSRP1* gene sequence were checked for their respective homologs within the HMP Database (http://www.hmpdacc.org) using a combination of the lenient and strict search criteria (maximum e-value cutoff of 1e-05 and 1e-50, respectively) and within NCBI database using BLASTx tool using a maximum e-value cutoff of 0.0.

### Data availability

SSU rRNA gene sequences have been submitted to the National Centre for Biotechnology Information (NCBI) Sequence Read Archive database, project number: PRJNA421267. The *pSR*6 & *pSR*7 DNA insert sequences were deposited under NCBI accession numbers of MG603294 & MG603295. SSU rRNA gene sequence data for the samples included in this study and *pSR*6, *pSR*7 sequences can also be accessed using the following link https://figshare.com/s/010669c3d83c30bf33c4.

## Electronic supplementary material

Supplementary File

## References

[1] Waditee R, Hibino T, Nakamura T, Incharoensakdi A, Takabe T (2002). Overexpression of a Na + /H + antiporter confers salt tolerance on a freshwater cyanobacterium, making it capable of growth in sea water. Proc. Natl. Acad. Sci. USA.

[2] Hillmann. F, Fischer RJ, Bahl H (2006). The rubrerythrin-like protein Hsp21 of *Clostridium acetobutylicum* is a general stress protein. Arch. In Microbiol..

[3] Roberts MF (2005). Organic compatible solutes of halotolerant and halophilic microorganisms. Saline Systems.

[4] Sakamoto T, Murata N (2002). Regulation of the desaturation of fatty acids and its role in tolerance to cold and salt stress. Curr. Opin. Microbiol..

[5] Zuleta LFG, Italiani VCS, Marques MV (2003). Isolation and characterization of NaCl-sensitive mutants of *Caulobactercrescentus*. Appl. Environ. Microbiol..

[6] Naughton LM, Blumerman SL, Carlberg M, Boyd EF (2009). Osmoadaptation among *Vibrio* species and unique genomic features and physiological responses of *Vibrio parahaemolyticus*. Appl. Environ. Microbiol..

[7] Klahn S, Marquardt DM, Rollwitz I, Hagemann M (2009). Expression of the *ggpPS* gene for glucosylglycerol biosynthesis from *Azotobacter vinelandii* improves the salt tolerance of *Arabidopsis thaliana*. J. Exp. Bot..

[8] Singh AH, Doerks T, Letunic I, Raes J, Bork P (2009). Discovering functional novelty in metagenomes: examples from light-mediated processes. J. Bacteriol..

[9] Singh J (2009). Metagenomics: concept, methodology, ecological inference and recent advances. Biotechnol. J..

[10] Hess M (2011). Metagenomic discovery of biomass-degrading genes and genomes from cow rumen. Science.

[11] Gupta S (2017). Systemic analysis of soil microbiome deciphers anthropogenic influence on soil ecology and ecosystem functioning. Int. J. Environ. Sci. Technol..

[12] Handelsman J (2004). Metagenomics: application of genomics to uncultured microorganisms. Microbiol. Mol. Biol. Rev..

[13] Chauhan NS, Nain S, Sharma R (2017). Identification of Arsenic Resistance Genes from Marine Sediment Metagenome. Indian J. Microbiol..

[14] Wang WL (2015). Application of metagenomics in the human gut microbiome. World. J. Gastroenterol..

[15] Yadav, M., Verma, M.K. & Chauhan, N.S. A review of metabolic potential of human gut microbiome in human nutrition. *Arch. Microbiol*. 10.1007/s00203-017-1459-x (2017). 10.1007/s00203-017-1459-x 29188341

[16] Feeney A, Sleator RD (2012). The human gut microbiome: the ghost in the machine. Future Microbiol..

[17] Sleator RD, Watson D, Hill C, Gahan CG (2009). The interaction between *Listeria monocytogenes* and the host gastrointestinal tract. Microbiol..

[18] Louis P, O’Byrne CP (2010). Life in the gut: microbial responses to stress in the gastrointestinal tract. Science Prog..

[19] Mondal AK (2017). Comparative Genomics of Host–Symbiont and Free-Living *Oceanobacillus* species. Genome Biol. Evol..

[20] Gerritsen J, Smidt H, Rijkers de Vos WM (2011). Intestinal microbiota in human health and disease: the impact of probiotics. Genes Nutr..

[21] Qin J (2010). A human gut microbial gene catalogue established by metagenomic sequencing. Nature.

[22] Culligan EP, Marchesi JR, Hill C, Sleator RD (2012). Mining of human gut microbiome for novel stress resistant genes. Gut Microbes.

[23] Culligan EP, Sleator RD, Marchesi JR, Hill C (2014). Metagenomic identification of novel salt tolerance gene from human gut microbiome which encodes a membrane protein with homology to a *brp/blh-* Family β-carotene 15, 15′-monoxygenase. PLoS One.

[24] Bhute S (2016). Molecular Characterization and Meta-Analysis of Gut Microbial Communities Illustrate Enrichment of *Prevotella* and *Megasphaera* in Indian Subjects. Front. Microbiol..

[25] Xu Y (2009). Crystal structure of the periplasmic region of MacB, a noncanonic ABC transporter. Biochemistry.

[26] Chimento DP, Kadner RJ, Wiener MC (2003). The *Escherichia coli* outer membrane cobalamin transporter BtuB: structural analysis of calcium and substrate binding, and identification of orthologous transporters by sequence/structure conservation. J. Mol. Biol..

[27] Sleator RD (2012). Proteins: form and function. Bioengineered Bugs.

[28] Sleator RD (2013). A Beginner’s Guide to Phylogenetics. MicrobEcol.

[29] Kumar J, Kumar M, Pandey R, Chauhan NS (2017). Physiopathology and management of gluten induced celiac disease. J. Food Sci..

[30] Dehingia M (2015). Gut bacterial diversity of the tribes of India and comparison with the worldwide data. Sci. Rep..

[31] De Filippo C (2010). Impact of diet in shaping gut microbiota revealed by a comparative study in children from Europe and rural Africa. Proc. Natl. Acad. Sci. USA.

[32] Vecchio MG (2014). Types of food and nutrient intake in India: a literature review. Indian J. Pediatr..

[33] Sleator RD, Gahan CG, Hill C (2003). A postgenomic appraisal of osmotolerance in *Listeria monocytogenes*. Appl. Environ. Microbiol..

[34] Hoffmann RF, McLernon S, Feeney A, Hill C, Sleator RD (2013). A single point mutation in the listerial *betL* sigma (A) -dependent promoter leads to improved osmo- and chill-tolerance and a morphological shift at elevated osmolarity. Bioengineered.

[35] Battesti A, Majdalani N, Gottesman S (2011). The RpoS-mediated general stress response in *Escherichia coli*. Ann. Rev. Microbiol..

[36] Chiang SM, Schellhorn HE (2012). Regulators of oxidative stress response genes in *Escherichia coli* and their functional conservation in bacteria. Arch. Biochem. Biophys..

[37] Celia H (2016). Structural insight into the role of the Ton complex in energy transduction. Nature.

[38] Wei B (2001). Molecular cloning of a *Bacteroides caccae* TonB-linked outer membrane protein identified by an inflammatory bowel disease marker antibody. Infect. Immun..

[39] Hall LM (2005). Sequence diversity and antigenic variation at the rag locus of *Porphyromonas gingivalis*. Infect. Immun..

[40] Cho KH, Salyers AA (2001). Biochemical analysis of interactions between outer membrane proteins that contribute to starch utilization by *Bacteroides thetaiotaomicron*. J. Bacteriol..

[41] Kumar J (2016). An Improved Methodology to Overcome Key Issues Associated with the Methods of Human Fecal Metagenomic DNA Extraction. Genomics Proteomics and Bioinformatics.

[42] Morowitz MJ (2011). Strain- resolved community genomic analysis of gut microbial colonization in premature infant. Proc. Natl. Acad. Sci. USA.

[43] Caporasoa JG (2011). Global patterns of 16S rRNA diversity at a depth of millions of sequences per sample. Proc. Natl. Acad. Sci. USA.

[44] Edgar RC (2010). Search and clustering orders of magnitude faster than BLAST. Bioinformatics.

[45] Lin A (2013). Distinct distal gut microbiome diversity and composition in healthy children from Bangladesh and the United States. PLoS One.

[46] Escobar JS, Klotz B, Valdes BE, Agudelo GM (2014). The gut microbiota of Colombians differs from that of Americans, Europeans and Asians. BMC Microbiol..

[47] Segata N (2011). Metagenomic biomarker discovery and explanation. Genome Biol..

[48] Chauhan NS, Ranjan R, Purohit HJ, Kalia VC, Sharma R (2009). Identification of genes conferring arsenic resistance to *Escherichia coli* from an effluent treatment plant sludge metagenomic library. FEMS Microbiol. Ecol..

[49] Sambrook, J. & Russel, D.W. Molecular cloning a laboratory manual (2001).

[50] Finn RD (2014). Pfam: the protein families database. Nucleic Acids Res..

[51] Von Mering C (2003). STRING: a database of predicted functional associations between proteins. Nucleic Acids Res..

[52] Zhou H, Zhou Y (2003). Predicting the topology of transmembrane helical proteins using mean burial propensity and a hidden-Markov-model-based method. Protein Sci..

[53] Tsirigos KD, Peters C, Shu N, Käll L, Elofsson A (2015). The TOPCONS web server for consensus prediction of membrane protein topology and signal peptides. Nucleic Acids Res..

[54] Webb AJ, Hosie AHF (2006). A Member of the Second Carbohydrate Uptake Subfamily of ATP-Binding Cassette Transporters is Responsible for Ribonucleoside Uptake in *Streptococcus* mutans. J. Bacteriol..

[55] Mirete S (2015). Salt resistance genes revealed by functional metagenomics from brines and moderate-salinity rhizosphere within a hypersaline environment. Front. Microbiol..

[56] Haardt M, Kempf B, Faatz E, Bremer E (1995). The osmoprotectant proline betaine is a major substrate for the binding-protein-dependent transport system ProU of *Escherichia coli* K-12. Mol. Gen. Genet..

