# Supplementary material for: Functional metagenomics identifies novel genes ABCTPP, TMSRP1 and TLSRP1 among human gut enterotypes
Source: Sci Rep. 2018 Jan 23;8:1397. doi: 10.1038/s41598-018-19862-5 (PMC5780487; doi:10.1038/s41598-018-19862-5)
Supplement: Supplementary file 1 — Supplementary File [file 41598_2018_19862_MOESM1_ESM.pdf]

Functional metagenomics identifies novel genes *ABCTPP*, *TMSRP1* and *TLSRP1* among human gut enterotypes

Authors

Manoj Kumar Verma[1,*], Vasim Ahmed[1,*], Shashank Gupta[1], Jitendra Kumar[1], Rajesh Pandey[2, 3], Vibha Mandhan[1], Nar Singh Chauhan[1,*]

* - These authors contributed equally

[1]*Department of Biochemistry, Maharshi Dayanand University, Rohtak, Haryana, India*

[2]*Ayurgenomics Unit-TRISUTRA, Council of Scientific and Industrial Research-Institute of Genomics and Integrative Biology, New Delhi, India*

[3]*Current affiliation: Mammalian Genetics Unit, MRC Harwell Institute, Harwell Science and Innovation Campus, Oxfordshire OX11 0RD, United Kingdom.*

**Corresponding Author*

Dr Nar Singh Chauhan,

Department of Biochemistry, Maharshi Dayanand University, Rohtak 124001, Haryana (India)

Email: nschauhan@mdurohtak.ac.in

Subject Category: Microbial population and community ecology

Running Title: Verma et al.,/Novel Osmotolerance genes from human gut Microbiome

Supplementary information

(A) Supplementary Tables

Table S1. Details of SSU rRNA gene sequence datasets generated in present study.

Sr. No	Sample ID	Number of Raw reads	NCBI Accession No.
**1**	**CC11**	67262	SRR6354880
**2**	**CC12**	34189	SRR6354881
**3**	**CC13**	30484	SRR6354878
**4**	**CC14**	25622	SRR6354879
**5**	**CC15**	18439	SRR6354876
**6**	**CC16**	28582	SRR6354877
**7**	**CC17**	26831	SRR6354874
**8**	**CC18**	28723	SRR6354875

Table S2. SSU rRNA gene sequence datasets used for comparative analysis in present study.

Country	Number of Samples	Database: accession numbers	SSU rRNA gene region amplified	References
Bangladesh	15	PRJNA173812 (SRR605700, SRR605701, SRR605702, SRR605703, SRR605704, SRR605708, SRR605709, SRR605710, SRR605711, SRR605739, SRR605740, SRR605741, SRR605742, SRR605743, SRR605744)	V1-V3	45
USA	15	PRJNA173812 (SRR605723, SRR605724, SRR605725, SRR605726, SRR605727, SRR605728, SRR605737, SRR605738, SRR605736, SRR605734, SRR605733, SRR605732, SRR605731, SRR605730, SRR605729)	V1-V3	45
In-house	8	This study	V1-V4	-
Columbia	15	ERP003466 (SRR605723, SRR605724, SRR605725, SRR605726, SRR605727, SRR605728, SRR605737, SRR605738, SRR605736, SRR605734, SRR605733, SRR605732, SRR605731, SRR605730, SRR605729)	V1-V3	46
India Tribes	17	MG-RAST id (4556487.3, 4553197.3, 4553198.3, 4565522.3, 4553287.3, 4553288.3, 4553289.3, 4553290.3, 4553291.3, 4553292.3, 4553363.3, 4565521.3, 4553355.3, 4553356.3, 4553357.3, 4553328.3, 4553329.3)	V3-V4	30

Table S3. Taxa significantly differentiated between the different populations identified by linear discriminant analysis coupled with effect size (LEfSe) using the default parameters.

TAXA	Country	P-VALUE
Bacilli	Bangladesh	4.99E-05
Bacteria.Firmicutes.Bacilli.Lactobacillales	Bangladesh	1.77E-05
Bacteria.Firmicutes.Bacilli.Lactobacillales.Leuconostocaceae	Bangladesh	1.12E-08
Bacteria.Firmicutes.Bacilli.Lactobacillales.Streptococcaceae	Bangladesh	0.006405049
Bacteria.Firmicutes.Bacilli.Lactobacillales.Streptococcaceae.Lactococcus	Bangladesh	7.58E-05
Bacteria.Firmicutes.Bacilli.Lactobacillales.Streptococcaceae.Lactococcus.OTUID_345575	Bangladesh	0.004158584
Bacteria.Firmicutes.Clostridia.Clostridiales.Clostridiaceae	Bangladesh	0.001732165
Bacteria.Firmicutes.Clostridia.Clostridiales.OTUID_313423	Bangladesh	9.73E-06
Bacteria.Firmicutes.Clostridia.Clostridiales.Ruminococcaceae.OTUID_192347	Bangladesh	0.001744752
Bacteria.Firmicutes.Erysipelotrichi	Bangladesh	0.000000002
Bacteria.Firmicutes.Erysipelotrichi.Erysipelotrichales	Bangladesh	0.000000002
Bacteria.Firmicutes.Erysipelotrichi.Erysipelotrichales.Erysipelotrichaceae	Bangladesh	0.000000002
Bacteria.Firmicutes.Erysipelotrichi.Erysipelotrichales.Erysipelotrichaceae._Eubacterium_	Bangladesh	1.06E-08
Bacteria.Firmicutes.Erysipelotrichi.Erysipelotrichales.Erysipelotrichaceae._Eubacterium_.biforme	Bangladesh	1.32E-09

.OTUID_193623		
Bacteria.Firmicutes.Erysipelotrichi.Erysipelotrichales.Erysipelotrichaceae.p_75_a5	Bangladesh	5.77E-07
Bacteria.Firmicutes.Erysipelotrichi.Erysipelotrichales.Erysipelotrichaceae.p_75_a5.OTUID_323200	Bangladesh	1.70E-07
Bacteria.Proteobacteria	Bangladesh	0.000659674
Bacteria.Proteobacteria.Gammaproteobacteria	Bangladesh	0.000899545
Bacteria.Proteobacteria.Gammaproteobacteria.Enterobacteriales	Bangladesh	0.000718328
Bacteria.Proteobacteria.Gammaproteobacteria.Enterobacteriales.Enterobacteriaceae	Bangladesh	0.000718328
Bacteria.Proteobacteria.Gammaproteobacteria.Enterobacteriales.Enterobacteriaceae.OTUID_197286	Bangladesh	0.001033581
Bacteria.Tenericutes	Bangladesh	0.000696221
Bacteria.Tenericutes.Mollicutes	Bangladesh	0.000231296
Bacteria.Tenericutes.Mollicutes.RF39	Bangladesh	0.000107779
Bacteria.Tenericutes.Mollicutes.RF39.OTUID_4308127	Bangladesh	3.74E-05
Bacteria.Tenericutes.Mollicutes.RF39.OTUID_569244	Bangladesh	0.000734829
Bacteria	Columbia	2.22E-05
Bacteria.Bacteroidetes.Bacteroidia.Bacteroidales.Bacteroidaceae.Bacteroides.OTUID_580629	Columbia	6.28E-07
Bacteria.Firmicutes	Columbia	0.000010501

Bacteria.Firmicutes.Clostridia	Columbia	0.000112961
Bacteria.Firmicutes.Clostridia.Clostridiales	Columbia	0.000120296
Bacteria.Firmicutes.Clostridia.Clostridiales.Lachnospiraceae	Columbia	2.87E-08
Bacteria.Firmicutes.Clostridia.Clostridiales.Lachnospiraceae._Ruminococcus_	Columbia	6.04E-08
Bacteria.Firmicutes.Clostridia.Clostridiales.Lachnospiraceae.Anaerostipes	Columbia	5.26E-07
Bacteria.Firmicutes.Clostridia.Clostridiales.Lachnospiraceae.Blautia	Columbia	0.000000144
Bacteria.Firmicutes.Clostridia.Clostridiales.Ruminococcaceae	Columbia	0.000144659
Bacteria.Firmicutes.Clostridia.Clostridiales.Ruminococcaceae.Faecalibacterium	Columbia	4.01E-10
Bacteria.Firmicutes.Clostridia.Clostridiales.Ruminococcaceae.Faecalibacterium.prausnitzii	Columbia	4.01E-10
Bacteria.Firmicutes.Clostridia.Clostridiales.Ruminococcaceae.Ruminococcus	Columbia	0.000002286
Bacteria.Verrucomicrobia	Columbia	2.32E-09
Bacteria.Verrucomicrobia.Verrucomicrobiae	Columbia	3.11E-11
Bacteria.Verrucomicrobia.Verrucomicrobiae.Verrucomicrobiales	Columbia	3.11E-11
Bacteria.Verrucomicrobia.Verrucomicrobiae.Verrucomicrobiales.Verrucomicrobiaceae	Columbia	3.11E-11
Bacteria.Verrucomicrobia.Verrucomicrobiae.Verrucomicrobiales.Verrucomicrobiaceae.Akkermansia	Columbia	3.11E-11
Bacteria.Verrucomicrobia.Verrucomicrobiae.Verrucomicrobiales.Verrucomicrobiaceae.Akkermansia.muciniphila	Columbia	3.11E-11

Bacteria.Verrucomicrobia.Verrucomicrobiae.Verrucomicrobiales.Verrucomicrobiaceae.Akkermansia.muciniphila.OTUID_593043	Columbia	0.00000357
Bacteria.Bacteroidetes	India	4.66E-08
Bacteria.Bacteroidetes.Bacteroidia	India	0.000000028
Bacteria.Bacteroidetes.Bacteroidia.Bacteroidales	India	0.000000028
Bacteria.Bacteroidetes.Bacteroidia.Bacteroidales.Bacteroidaceae.Bacteroides.fragilis.OTUID_351231	India	0.000000206
Bacteria.Bacteroidetes.Bacteroidia.Bacteroidales.Prevotellaceae	India	1.41E-09
Bacteria.Bacteroidetes.Bacteroidia.Bacteroidales.Prevotellaceae.Prevotella	India	1.41E-09
Bacteria.Bacteroidetes.Bacteroidia.Bacteroidales.Prevotellaceae.Prevotella.copri	India	1.68E-09
Bacteria.Bacteroidetes.Bacteroidia.Bacteroidales.Prevotellaceae.Prevotella.copri.OTUID_545061	India	0.00000001
Bacteria.Bacteroidetes.Bacteroidia.Bacteroidales.Prevotellaceae.Prevotella.copri.OTUID_568118	India	6.86E-12
Bacteria.Bacteroidetes.Bacteroidia.Bacteroidales.Prevotellaceae.Prevotella.copri.OTUID_589329	India	3.75E-09
Bacteria.Firmicutes.Bacilli.Lactobacillales.Lactobacillaceae	India	1.61E-07
Bacteria.Firmicutes.Bacilli.Lactobacillales.Lactobacillaceae.Lactobacillus	India	1.61E-07
Bacteria.Firmicutes.Bacilli.Lactobacillales.Lactobacillaceae.Lactobacillus.ruminis	India	6.18E-07
Bacteria.Firmicutes.Bacilli.Lactobacillales.Lactobacillaceae.Lactobacillus.ruminis.OTUID_178213	India	6.18E-07

Bacteria.Firmicutes.Clostridia.Clostridiales.Lachnospiraceae.Roseburia	India	9.85E-05
Bacteria.Firmicutes.Clostridia.Clostridiales.Ruminococcaceae.Faecalibacterium.prausnitzii.OTUID_370287	India	2.43E-10
Bacteria.Proteobacteria.Betaproteobacteria	India	1.35E-06
Bacteria.Proteobacteria.Betaproteobacteria.Burkholderiales	India	3.22E-07
Bacteria.Proteobacteria.Betaproteobacteria.Burkholderiales.Alcaligenaceae	India	5.67E-08
Bacteria.Proteobacteria.Betaproteobacteria.Burkholderiales.Alcaligenaceae.Sutterella	India	5.67E-08
Bacteria.Proteobacteria.Betaproteobacteria.Burkholderiales.Alcaligenaceae.Sutterella.OTUID_1974536	India	6.76E-05
Bacteria.Actinobacteria	Indian_Tribes	0.000000001
Bacteria.Bacteroidetes.Bacteroidia.Bacteroidales._Paraprevotellaceae_	Indian_Tribes	2.57E-08
Bacteria.Bacteroidetes.Bacteroidia.Bacteroidales._Paraprevotellaceae_._Prevotella_	Indian_Tribes	5.35E-08
Bacteria.Bacteroidetes.Bacteroidia.Bacteroidales.Prevotellaceae.Prevotella.copri.OTUID_173565	Indian_Tribes	1.05E-11
Bacteria.Bacteroidetes.Bacteroidia.Bacteroidales.Prevotellaceae.Prevotella.copri.OTUID_326482	Indian_Tribes	1.05E-12
Bacteria.Bacteroidetes.Bacteroidia.Bacteroidales.Prevotellaceae.Prevotella.copri.OTUID_346938	Indian_Tribes	3.95E-12
Bacteria.Bacteroidetes.Bacteroidia.Bacteroidales.Prevotellaceae.Prevotella.copri.OTUID_527941	Indian_Tribes	1.06E-11
Bacteria.Bacteroidetes.Bacteroidia.Bacteroidales.Prevotellaceae.Prevotella.copri.OTUID_530653	Indian_Tribes	3.88E-11
Bacteria.Bacteroidetes.Bacteroidia.Bacteroidales.Prevotellaceae.Prevotella.copri.OTUID_588929	Indian_Tribes	6.26E-12

Bacteria.Bacteroidetes.Bacteroidia.Bacteroidales.Prevotellaceae.Prevotella.stercorea	Indian_Tribes	5.53E-07
Bacteria.Firmicutes.Clostridia.Clostridiales.Lachnospiraceae.OTUID_367909	Indian_Tribes	3.05E-12
Bacteria.Proteobacteria.Gammaproteobacteria.Aeromonadales	Indian_Tribes	4.43E-09
Bacteria.Proteobacteria.Gammaproteobacteria.Aeromonadales.Succinivibrionaceae	Indian_Tribes	2.96E-10
Bacteria.Proteobacteria.Gammaproteobacteria.Aeromonadales.Succinivibrionaceae.Succinivibrio	Indian_Tribes	2.96E-10
Bacteria.Proteobacteria.Gammaproteobacteria.Aeromonadales.Succinivibrionaceae.Succinivibrio.OTUID_524117	Indian_Tribes	1.31E-10
Bacteria.Bacteroidetes.Bacteroidia.Bacteroidales._Odoribacteraceae_	USA	4.06E-06
Bacteria.Bacteroidetes.Bacteroidia.Bacteroidales.Bacteroidaceae	USA	0.000000002
Bacteria.Bacteroidetes.Bacteroidia.Bacteroidales.Bacteroidaceae.Bacteroides	USA	0.000000002
Bacteria.Bacteroidetes.Bacteroidia.Bacteroidales.Bacteroidaceae.Bacteroides.eggerthii	USA	6.57E-06
Bacteria.Bacteroidetes.Bacteroidia.Bacteroidales.Bacteroidaceae.Bacteroides.fragilis	USA	4.49E-07
Bacteria.Bacteroidetes.Bacteroidia.Bacteroidales.Bacteroidaceae.Bacteroides.fragilis.OTUID_183603	USA	6.02E-08
Bacteria.Bacteroidetes.Bacteroidia.Bacteroidales.Bacteroidaceae.Bacteroides.OTUID_171559	USA	0.000587166
Bacteria.Bacteroidetes.Bacteroidia.Bacteroidales.Bacteroidaceae.Bacteroides.OTUID_199716	USA	1.96E-06
Bacteria.Bacteroidetes.Bacteroidia.Bacteroidales.Bacteroidaceae.Bacteroides.OTUID_359538	USA	0.001469293
Bacteria.Bacteroidetes.Bacteroidia.Bacteroidales.Bacteroidaceae.Bacteroides.OTUID_550814	USA	1.80E-05

Bacteria.Bacteroidetes.Bacteroidia.Bacteroidales.Bacteroidaceae.Bacteroides.OTUID_560336	USA	3.84E-08
Bacteria.Bacteroidetes.Bacteroidia.Bacteroidales.Bacteroidaceae.Bacteroides.uniformis	USA	1.42E-09
Bacteria.Bacteroidetes.Bacteroidia.Bacteroidales.Bacteroidaceae.Bacteroides.uniformis.OTUID_348027	USA	4.31E-12
Bacteria.Firmicutes.Clostridia.Clostridiales.Veillonellaceae	USA	1.49E-09
Bacteria.Firmicutes.Clostridia.Clostridiales.Veillonellaceae.Dialister	USA	0.000267425
Bacteria.Firmicutes.Clostridia.Clostridiales.Veillonellaceae.Dialister.OTUID_201364	USA	0.003031316
Bacteria.Firmicutes.Clostridia.Clostridiales.Veillonellaceae.Dialister.OTUID_403701	USA	0.031807823
Bacteria.Firmicutes.Clostridia.Clostridiales.Veillonellaceae.Phascolarctobacterium	USA	0.003149166
Bacteria.Firmicutes.Clostridia.Clostridiales.Veillonellaceae.Phascolarctobacterium.OTUID_556835	USA	0.020824317

Table S4. Metadata information of the individual engaged in the present study.

Sample	Sex	Any associated Disease	Any antibiotics/ Probiotics intake	Diet and smoking status
CC11	Male	No	No	Milk, Diary products, wheat bread, vegetables, fruits, egg and Non Smoker
CC12	Female	No	No	Milk, Diary products, wheat bread, vegetables, fruits, egg and Non Smoker
CC13	Male	No	No	Milk, Diary products, wheat bread, vegetables, fruits, egg and Non Smoker
CC14	Male	No	No	Milk, Diary products, wheat bread, vegetables, fruits, egg and Non Smoker
CC15	Female	No	No	Milk, Diary products, wheat bread, vegetables, fruits, egg and Non Smoker
CC16	Male	No	No	Milk, Diary products, wheat bread, vegetables, fruits, egg and Non Smoker
CC17	Female	No	No	Milk, Diary products, wheat bread, vegetables, fruits, egg and Non Smoker
CC18	Female	No	No	Milk, Diary products, wheat bread, vegetables, fruits, egg and Non Smoker

Table S5. Oligonucleotide sequence used in the present study.

Sr. No	Primer ID	Primer sequence (5'-3')	Length (base)	Source or reference
1.	ORF2P+Fwd	ATCCGAATTCAACTCTGAGATATTG	25	Present work
2.	ORF2 Rev	GCGAAGCTTCTACAACCGCGGAAC	24	Present work
3.	C1 Fwd	AGTGAATTCATGGAAGAGAAAGACCTG	27	Present work
4.	C1 Rev	GCCAAGCTTTTATTCGTCTCTCATTGC	27	Present work
5.	EZ-Tn5^{TM} FP	ACCTACAACAAAGCTCTCATCAACC	25	Present work
6.	EZ-Tn5^{TM} RP	GCAATGTAACATCAGAGATTTTGAG	25	Present work
7.	psr6fwd	ATCCGAATTCATGGAAGAGAAAGACCTG	28	Present work
8.	psr6rev	CGCAAGCTTTTCGTCTCTCATTGCATC	27	Present work
9.	Profwd	CCGGTACCAACTCTGAGATATTGACTTG	28	Present work
10.	Pro1rev	CCTGGTACCCATTTCCAGGTAGATTTCTG	29	Present work
11.	Pro2rev	CCTGGTACCCTTCCAGATGCTCGACAATC	29	Present work
12.	Pro3fwd	CCGGTACCATTTCATAAAGTTCAATGTTC	29	Present work
13.	Pro4fwd	CCGGTACCAATGTTCAAATCTC	22	Present work
14.	SSUrRNA fwd	CCATCTCATCCCTGCGTGTCTCCGACTCAGA CGAGTGCGTGAGTTGATCCTGGCTCAG	58	42
15.	SSUrRNA rev	CCTATCCCCTGTGTGCCTTGGCAGTCT CAGGACTACCAGGGTATCTAA	48	42

(B) Supplementary figure

Figure S1. Growth of SR6 (●), SR7 (▲) and *E. coli* (DH10B) host strain carrying empty plasmid vector (■) in **(a)** LB broth supplemented with 3.0% NaCl (w/v), **(b)** LB broth supplemented with 5% KCl (w/v), and **(c)** LB broth. Each point in graph is the mean of three different replicate experiments, each performed in triplicate.

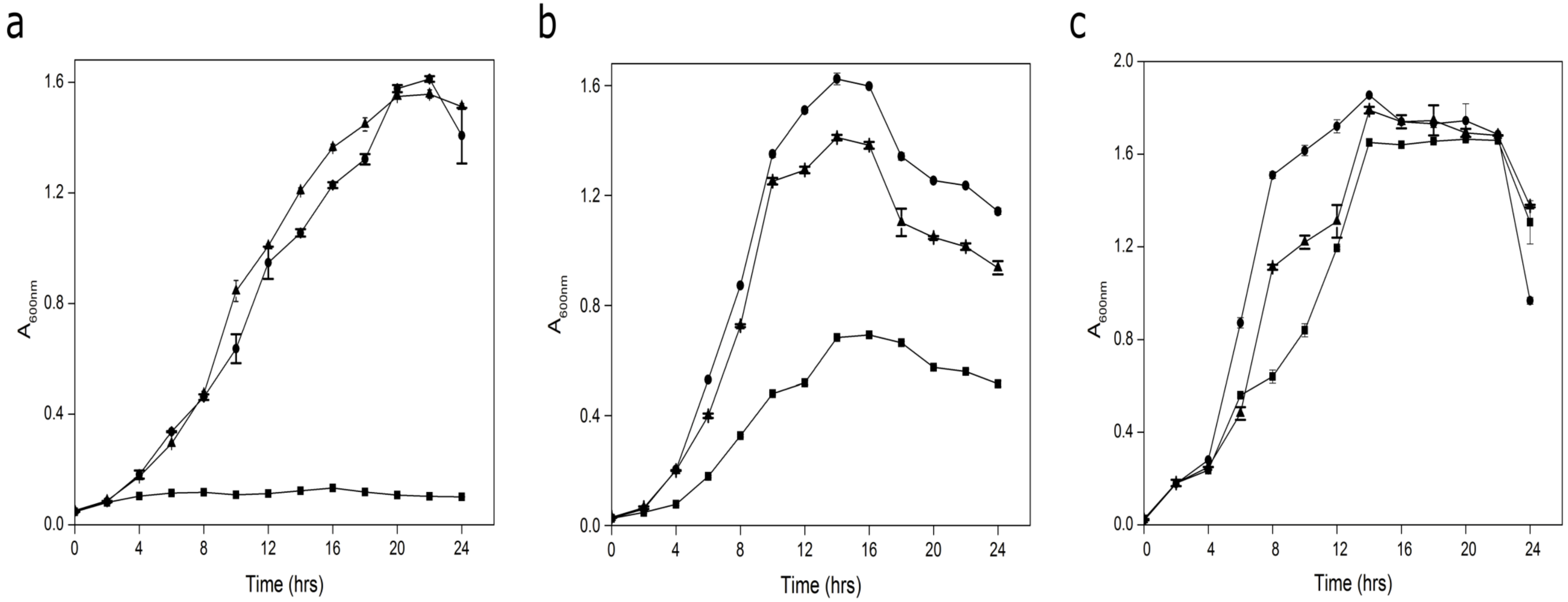

Figure S2. Minimum inhibitory concentration analysis of SR6 (●), SR7 (▲) and *E. coli* (DH10B) host strain carrying empty plasmid vector (■) in **(a)** LB broth supplemented with different concentrations of NaCl (*P*= 0.0158; *P*= 0.0344) **(b)** LB broth supplemented with different concentrations of KCl (*P*= 0.0099; *P*= 0.0088). Each point in graph is the mean of three different replicate experiments, each performed in triplicate.

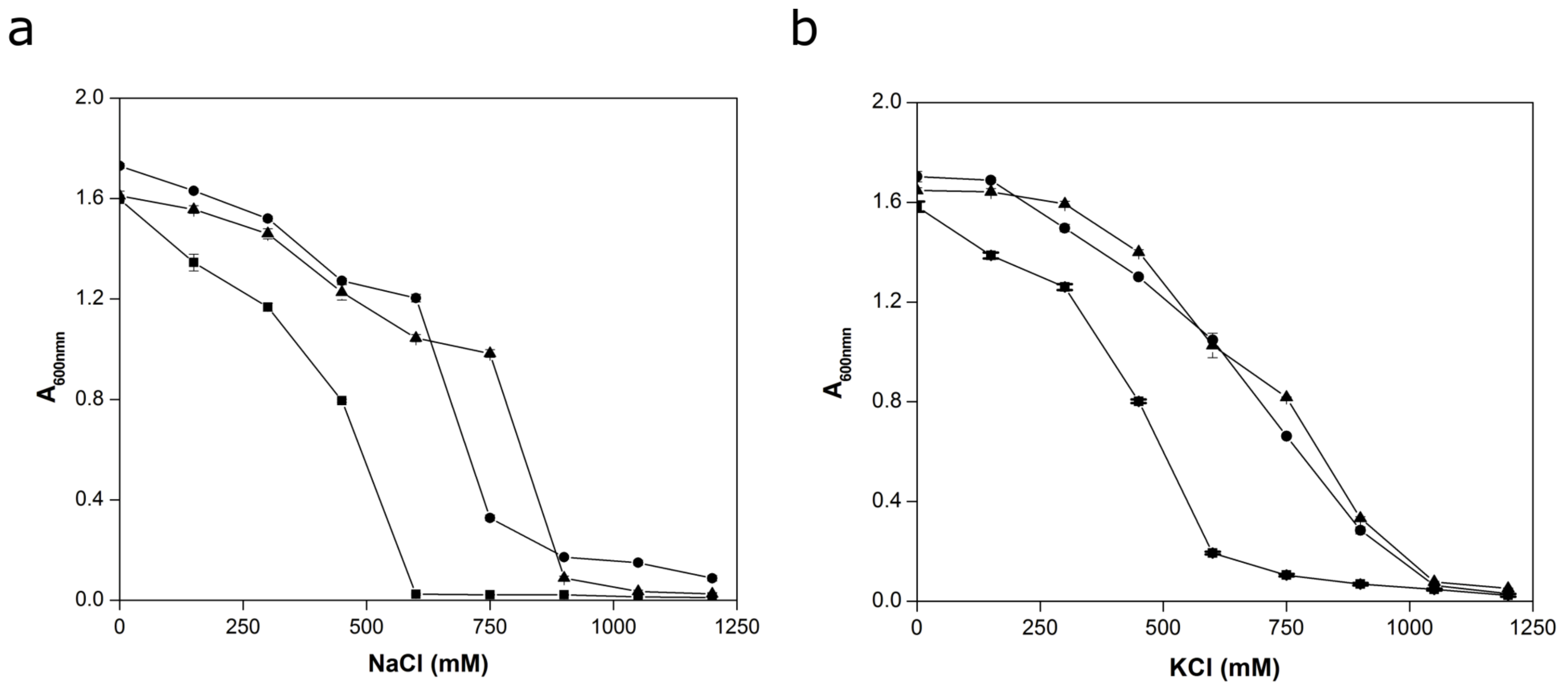

Figure S3. Growth of SR6C3 (▲) and *E. coli* (DH10B) host strain carrying empty plasmid vector (■) in **(a)** LB broth supplemented with 4.0% NaCl (w/v) **(b)** LB broth supplemented with 5% KCl (w/v). Each point in graph is the mean of three different replicate experiments, each performed in triplicate.

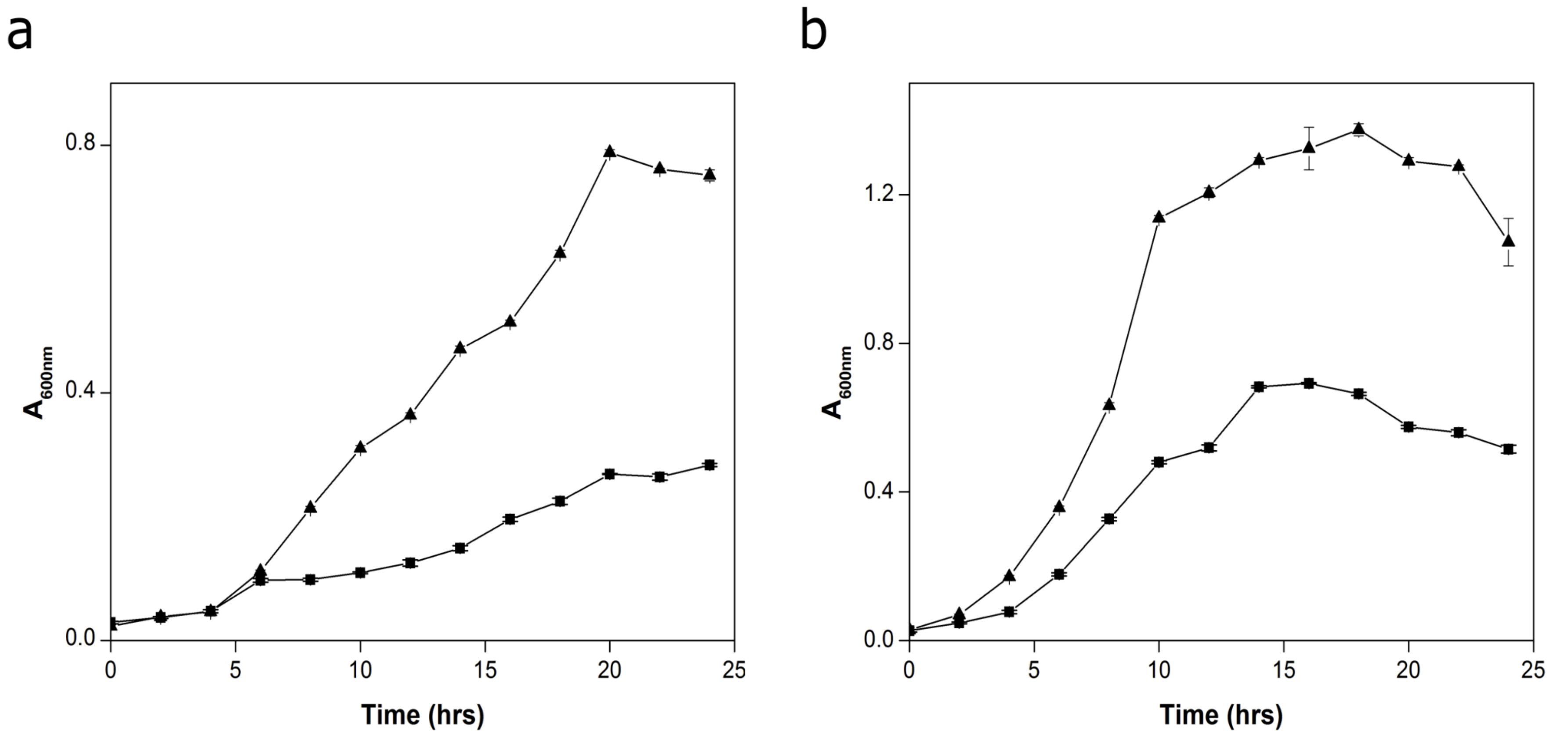

Figure S4. Promoter constructs map of *TMSRP1* gene.

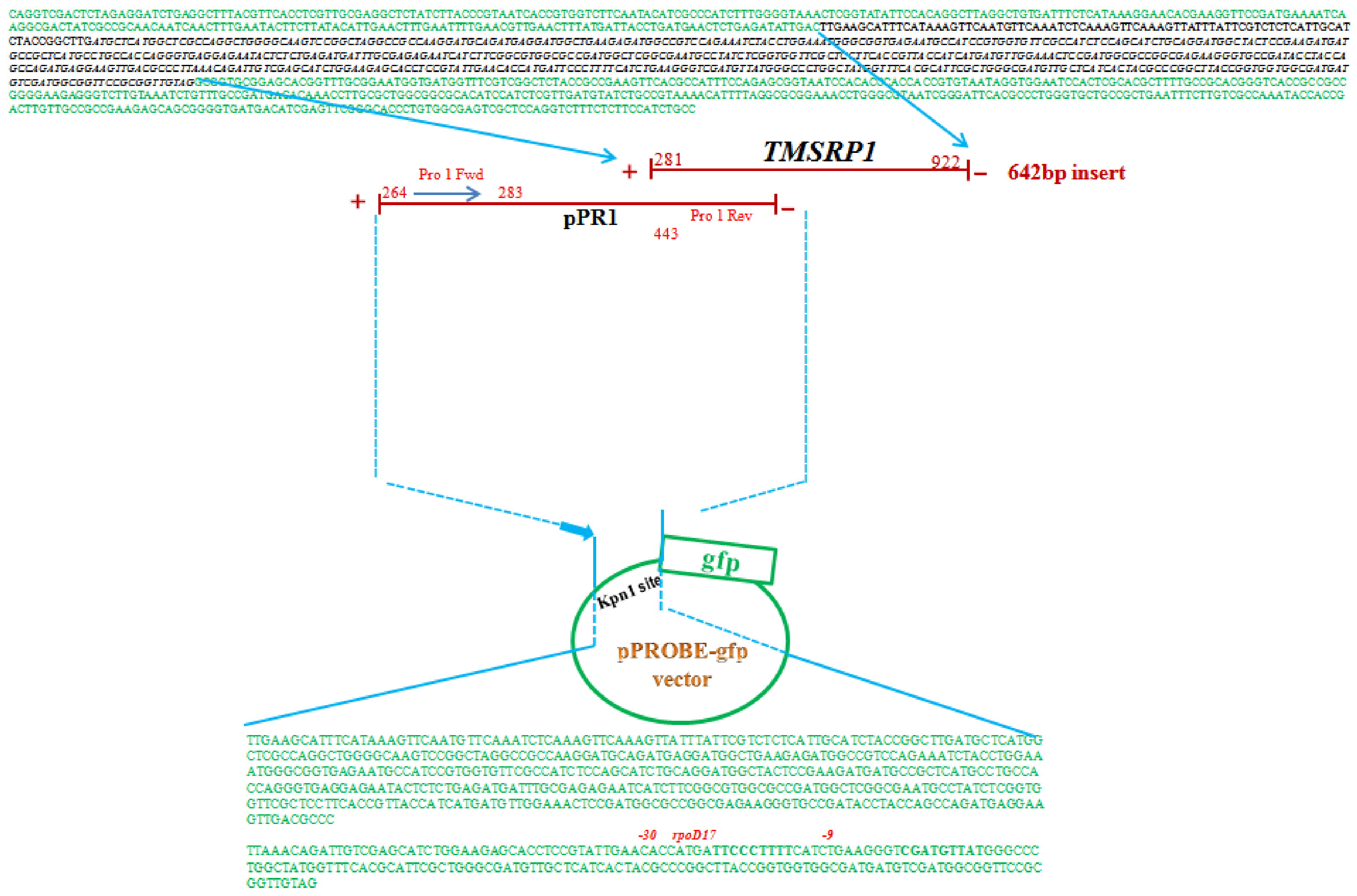

Figure S5. *TMSRP1*, *ABCTPP* and *TLSRP1* homologs identified when BLAST searched against Human Microbiome Project (HMP) datasets from 16 body sites at maximum e-value cut-off of ((A) 1e-50 and (B)1e-05) **(a), (b)** and **(c)**. Abundance of *TMSRP1*, *ABCTPP* and *TLSRP1* among various life forms **(d), (e)** and **(f)**.

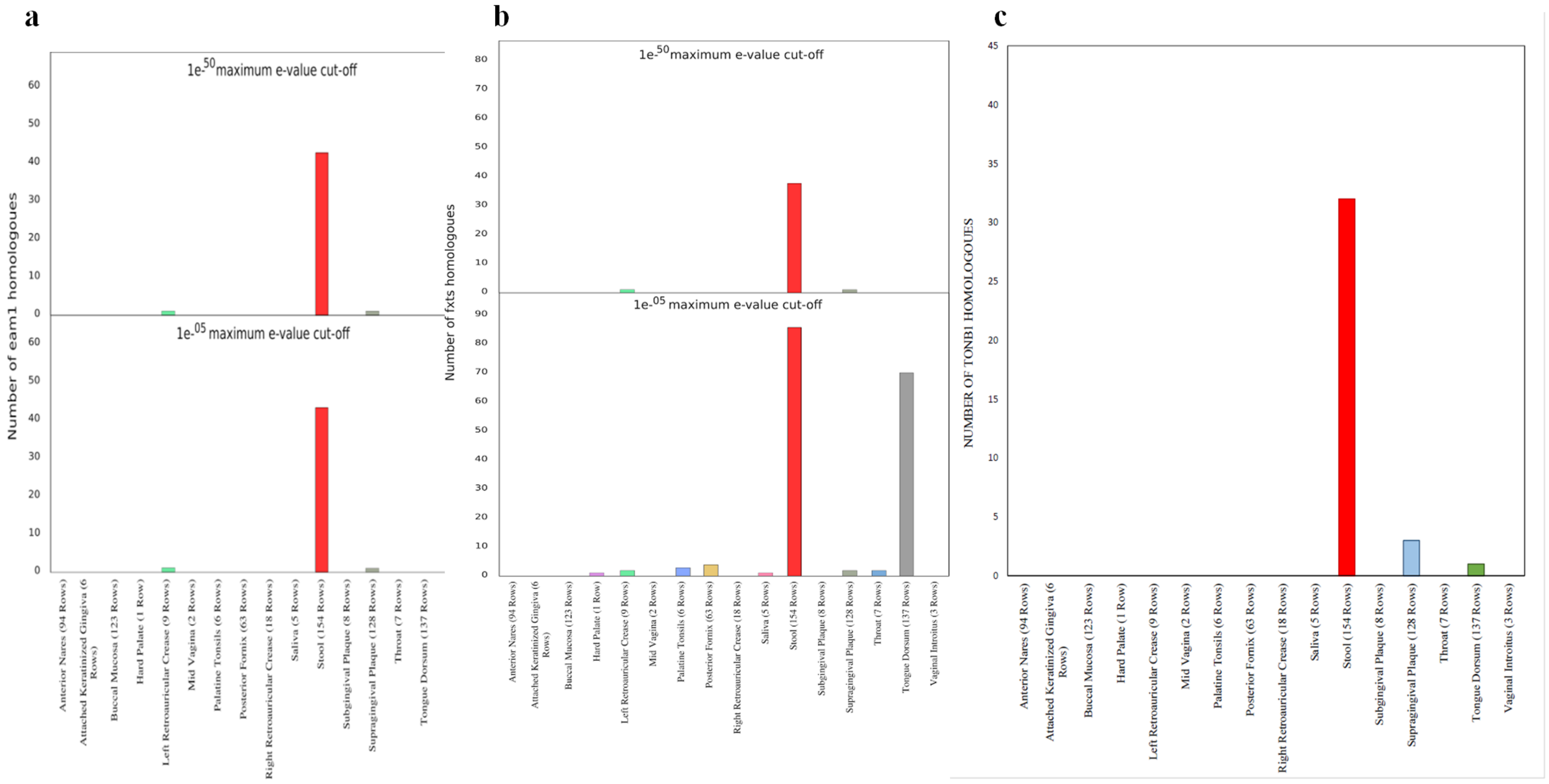

d

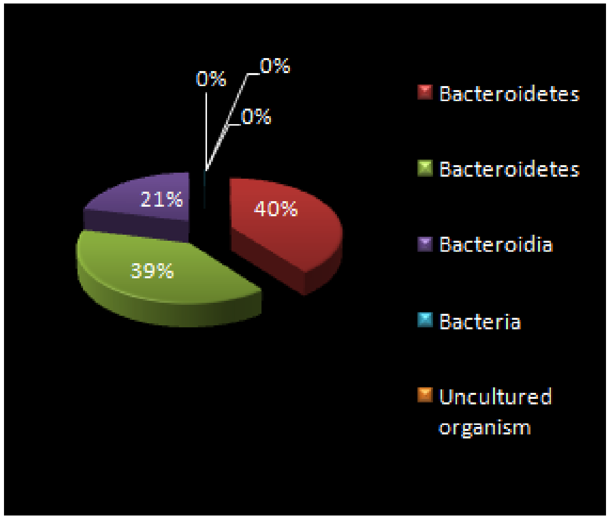
0%
0%
0%
21%
40%
39%
Bacteroidetes
Bacteroidetes
Bacteroidia
Bacteria
Uncultured organism

e

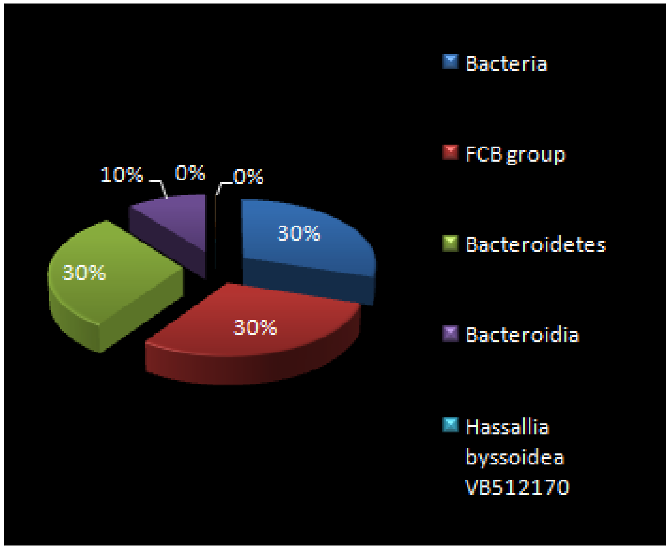
10%
0%
0%
30%
30%
30%
Bacteria
FCB group
Bacteroidetes
Bacteroidia
Hassallia byssoidea VB512170

f

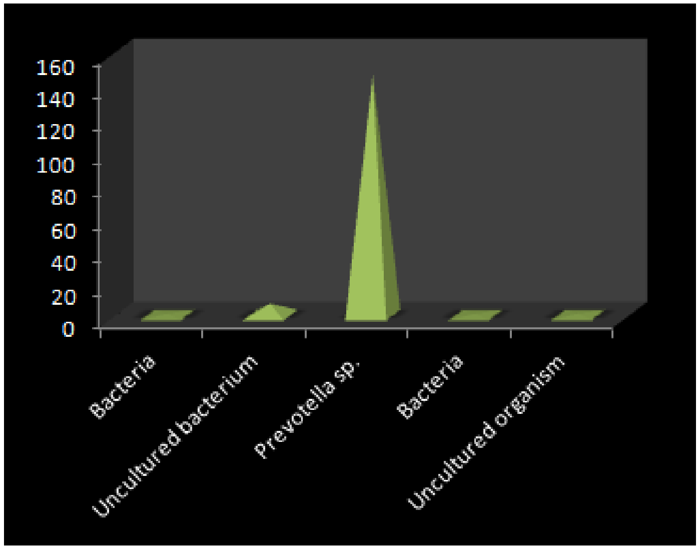
160
140
120
100
80
60
40
20
0
Bacteria
Uncultured bacterium
Prevotella sp.
Bacteria
Uncultured organism
